# Water-Soluble Quaternary and Protonable Basic Chitotriazolans: Synthesis by Click Chemistry Conversion of Chitosan Azides and Investigation of Antibacterial Activity

**DOI:** 10.3390/jfb15030063

**Published:** 2024-03-05

**Authors:** Sankar Rathinam, Romano Magdadaro, Martha Á. Hjálmarsdóttir, Már Másson

**Affiliations:** 1 Faculty of Pharmaceutical Sciences, School of Health Sciences, University of Iceland, Hofsvallagata 53, IS-107 Reykjavík, Iceland; sankar@hi.is (S.R.); c18434796@mytudublin.ie (R.M.); 2 Department of Biomedical Science, Faculty of Medicine, School of Health Sciences, University of Iceland, Hringbraut 31, IS-101 Reykjavík, Iceland; hjalmars@hi.is

**Keywords:** chitosan, click chemistry, triazole, antibacterial activity

## Abstract

The azide transfer reaction and copper(I)-catalyzed alkyne-azide cycloaddition (CuAAC) can be used to convert the amino groups in chitosan to triazole 1,2,3-moieties. The resulting polymer has been named chitotriazolan. This synthesis was performed with six different quaternary ammonium alkynes and three amine alkynes to obtain a series of nine water-soluble chitotriazolan derivatives. The structure and complete conversion of the azide were confirmed by FT-IR and proton NMR spectroscopy. The derivatives were investigated for antibacterial activity against *S. aureus*, *E. faecalis*, *E. coli*, and *P. aeruginosa.* The activity of the quaternized chitotriazolan derivatives varied depending on the structure of the quaternary moiety and the species of bacteria. The basic protonable derivatives were less active or inactive against the bacteria.

## 1. Introduction

Chitosan is a linear cationic polysaccharide that is composed of randomly distributed *β*-(1-4)-linked D-glucosamine and *N*-acetylated-D-glucosamine residues. It can be obtained by the *N*-deacetylation of chitin [1,2,3,4]. Chitosan and its derivatives are attractive biopolymers for various biomedical applications because they are biodegradable and biocompatible and have low toxicity. However, the application of chitosan is limited due to its low solubility at physiological pH. Consequently, there has been significant interest in the chemical modification of chitosan to obtain more soluble derivatives with enhanced biological properties. Most reported chitosan derivatives have been prepared by reactions with aldehydes and ketones to generate Schiff’s bases [5,6,7], acylation [8,9], alkylation [10,11,12], the chelation of metals [13,14], sulfonation [15,16], carboxymethylation [17,18,19], or free-radical grafting [20]. However, these standard methods sometimes require harsh conditions that affect the degree of polymerization [21], and it may be difficult to achieve both selective modification and a high degree of substitution.

In 2001, Sharpless and his co-workers introduced the concept of “click chemistry” to describe “spring-loaded” exothermic reactions that are versatile and modular, give high yields, and do not generate harmful by-products [22]. The copper-catalyzed azide–alkyne cycloaddition (CuAAC) reaction between terminal alkynes and azides has become the most widely used click chemistry reaction in the past two decades [23,24,25]. Although click chemistry reactions were originally designed to facilitate low-molecular-weight drug discovery, they are now used in broad areas of chemistry, such as material sciences [26,27], nanotechnology [28,29], and the bioconjugation and modification of DNA, natural products, and polymers [30,31]. This includes the click chemistry-based conjugation and modification of chitosan. However, before the CuAAC click reaction, chitosan has to be modified to introduce an azide functional group or a terminal alkyne backbone. This has been performed by (*i*) introducing an azide group or alkyne at chitosan’s *C*-6 hydroxyl in a multistep synthesis including protection and deprotection steps [32,33], (*ii*) introducing an azide functional group or alkyne onto the terminal of a spacer group introduced by *N*-acylation [34,35], and (*iii*) converting the chitosan amino group to an azide functional group via a diazo transfer reaction, followed by CuAAC reactions. Chitosan amine-to-azide conversion involves only a two-step synthesis under mild conditions, producing a quantitative yield and avoiding a reduction in the degree of polymerization [36,37,38].

The CuAAC reaction has also been used to conjugate the antimicrobial anoplin peptide to chitosan, and the antimicrobial peptide chitosan–triazole exhibited good antibacterial activity [35]. This conjugate exhibited improved antimicrobial activity and reduced toxicity relative to the parent peptide and polymer. Ultrasound in combination with copper wire was used to promote the cycloaddition of a quaternary ammonium alkyne to chitosan hydroxypropyl azide [39]. The same procedure was used to synthesize triazole betaine ester chitosan derivatives, which were formulated into nanoparticles with antibacterial activity and the ability to be used for in-vitro transfection [40].

The amino group of chitosan has been protected by a phthaloyl moiety, and the C-6 hydroxyl group has been converted to azide in a multistep synthesis [32,41,42,43,44,45,46,47]. The amino group of chitosan has also been *N*,*N*,*N*-trimethylated, and the *C*-6 hydroxyl group has been converted to azide, followed by a click reaction [45,48,49]. *O*-alkyne derivatives of trimethyl chitosan were obtained via a reaction with propargyl bromide under alkaline conditions, and then a click reaction was performed to give *N*,*N*,*N*-trimethyl-*N*-(2-azido)-ethyl ammonium bromide derivatives [50].

We have used imidazole sulfonyl azide HCl salt to convert chitosan to chitosan azide and DMSO as a solvent for the CuAAC reaction to achieve the first quantitative conversion of the free amino groups in chitosan to obtain water-soluble chitotriazolan derivatives with improved antibacterial activity relative to unmodified chitosan [37]. This procedure has also been used to convert the free amino groups of various *N*-alkyl and *N*-acyl chitosan derivatives and obtain water-soluble “mixed” chitotriazolans [51]. Chitosan triazole derivatives were modified by Wolff cyclocondensation between the chitosan amine group and *α*-diazo-*β*-oxoamides [52]. Chitosan amine and unsubstituted acetylene were converted to triazole moieties on chitosan by a click reaction [53].

This procedure was also used in the current study to convert chitosan to chitotriazolan derivatives with quaternary ammonium and protonatable amino moieties. The purpose of this work was to demonstrate the utility of the general applicability of the procedure for the fast and efficient synthesis of chitotriazolan derivatives with diverse structures and to allow a detailed study of the structure–activity relationship. Chitosan was converted to chitosan azide and reacted with various alkynes using a CuAAC reaction to give nine different chitotriazolan derivatives. These derivatives were characterized by IR and proton NMR spectroscopy and gel permeation chromatography to determine their molecular weights. These derivatives exhibited improved water solubility compared to unmodified chitosan and were evaluated for their antibacterial activity against four Gram-positive and Gram-negative bacteria.

## 2. Materials and Methods

### 2.1. Materials

Chitosan (TM3623, degree of deacetylation 82% and MW 269.5 kDa) was provided by Primex ehf, Siglufjördur, Iceland. All reagent-grade chemicals were purchased from Sigma Aldrich (Heidenheim, Germany): imidazole, sodium azide, sulfuryl chloride, acetyl chloride, hydrochloric acid, sodium bicarbonate, copper sulfate (II) pentahydrate, sodium ascorbate, propargyl bromide, trimethylamine, triethylamine, triethanolamine, diethanolamine, piperazine, *N*-methylpiperazine, 1,4-dimethylpiperazine, pyridine, 1-methylimidazole, potassium carbonate, di-*tert*-butyl-dicarbonate, cesium carbonate, sodium sulfate, and trifluoracetic acid. All solvents, namely, dimethyl sulfoxide (DMSO), acetone, methanol, ethanol, toluene, dichloromethane, ethyl acetate, isopropyl alcohol, and acetonitrile, were also obtained from Sigma Aldrich. De-ionized water was prepared using a Milli-Q™ filtration system (MerkMillipore, MA, USA). Dialysis membranes (RC, Spectra/Por, MW cutoff 3500 Da 45 mm) were purchased from Spectrum^®^ Laboratories Inc. (Rancho Dominguez, Los Angeles, CA, USA).

### 2.2. Experimental Methods

#### 2.2.1. Synthesis of Chitosan Azide 

We followed a synthetic procedure described in our recently published study [37]. To summarize, 500 mg of the chitosan derivatives was dissolved in a 0.1 M HCl solution (40 mL). Subsequently, one equivalent of NaHCO_3_ was added to the solution, which was then vigorously stirred for 30 min. Following this, imidazole sulfonyl azide hydrochloride (1 equiv) and NaHCO_3_ (10 equiv) were slowly added to the mixture. To continue the reaction, a solution of CuSO_4_ 5H_2_O in 2 mL of water and 10 mL of methanol was added, and the resulting reaction mixture was stirred at room temperature for 24 h. Finally, the resulting material was precipitated using acetone. The precipitate was then filtered and washed with excess water and acetone, after which the obtained product was dried. The presence of the azide group was confirmed by IR spectroscopy, specifically at a wavenumber of 2109 cm^−1^.

#### 2.2.2. Synthesis of Different Alkynes (**1a**–**9a**)

*N*,*N*,*N*-trimethylprop-2-yn-1-aminium (**1a**), *N*,*N*,*N*-tris(2-hydroxyethyl)prop-2-yn-1-aminium (**3a**), 1,4-dimethyl-1-(prop-2-yn-1-yl)piperazin-1-ium (**4a**), and 2,2′-(prop-2-yn-1-ylazanediyl)bis(ethan-1-ol) (**9a**) were synthesized according to our previous method [37]. Other alkynes were synthesized by following previously reported procedures. These were *N*,*N*,*N*-triethylprop-2-yn-1-aminium (**2a**) and 1-methyl-3-(prop-2-yn-1-yl)-1H-imidazol-3-ium (**6a**) [54], 1-(prop-2-yn-1-yl)pyridin-1-ium (**5a**) [55], 1-methyl-4-(prop-2-yn-1-yl)piperazine (**7a**), and 1-(prop-2-yn-1-yl)piperazine (**8a**) [56,57].

#### 2.2.3. General Synthesis Procedure for Chitotriazolan Derivatives **1**–**9**

Chitosan azide was converted to chitotriazoan derivatives following a procedure reported in our previous publication [37]. Chitosan azide (1 equiv) was dissolved in DMSO at 50 °C. Then, a solution of CuSO_4_ 5H_2_O (2.5 mL water), sodium ascorbate (2.5 mL water), and the alkyne (**1a**–**9a**) was added to the reaction mixture under a nitrogen atmosphere. The reaction mixture was stirred at 50 °C for 48 h. Following this, the resulting material was dialyzed against water for four days, employing ion exchange with 5% NaCl. Finally, the product was freeze-dried. The successful conversion was confirmed by the complete disappearance of the azide peak at 2109 cm^−1^ in the FT-IR spectrum. 

Procedure for chitotriazolan derivative (**1**): Chitosan azide (200 mg, 1.07 mmol) in DMSO (15 mL), CuSO_4_ 5H_2_O (34 mg, 0.139 mmol), sodium ascorbate (106 mg, 0.534 mmol), and *N*-propargyl-*N*,*N*,*N*-trimethylammonium bromide (**1a**) (523 mg, 5.34 mmol). Yield: 82%. ^1^H NMR (400 MHz, D_2_O): *δ* 2.08 (N-COCH_3_), 2.90 (H6′), 3.20 [H6, *N*(CH_3_)_3_], 3.52 (H5), 3.78 (H4), 4.44 (H3), 4.58 (H2), 4.77 (triazole CH_2_ merged with D_2_O peak), 5.18 (H1), 8.59 (triazole CH).

Procedure for chitotriazolan derivative (**2**): Chitosan azide (200 mg, 1.07 mmol) in DMSO (15 mL), CuSO_4_ 5H_2_O (34 mg, 0.139 mmol), sodium ascorbate (106 mg, 0.534 mmol), and *N*,*N*,*N*-triethylprop-2-yn-1-aminium (**2a**) (749 mg, 5.34 mmol). Yield: 91%. ^1^H NMR (400 MHz, D_2_O): *δ* 1.45 [*N*(Ethyl-CH_3_)_3_], 2.08 (N-COCH_3_), 2.89 (H6′), 3.11 (H6), 3.33 [*N*(CH_2_)_3_], 3.49 (H5), 3.77 (H4), 4.42 (H3), 4.52 (H2), 4.66 (triazole CH_2_ merged with D_2_O peak), 5.17 (H1), 8.57 (triazole CH).

Procedure for chitoriazolan derivative (**3**): Chitosan azide (200 mg, 1.07 mmol) in DMSO (15 mL), CuSO_4_ 5H_2_O (34 mg, 0.139 mmol), sodium ascorbate (106 mg, 0.534 mmol), and *N*,*N*,*N*-tris(2-hydroxyethyl)prop-2-yn-1-aminium (**3a**) (1.005 g, 5.34 mmol). Yield: 80%. ^1^H NMR (400 MHz, D_2_O): *δ* 2.08 (N-COCH_3_), 2.88 (H6′), 3.12 (H6), 3.51 (H5), 3.68 [(H4), [(*N*(CH_2_-CH_2_OH)_3_] 4.21 [(*N*(CH_2_-CH_2_OH)_3_] 4.41 (H3), 4.56 (H2), 5.03 (triazole CH_2_), 5.14 (H1), 8.56 (triazole CH).

Procedure for chitotriazolan derivative (**4**): Chitosan azide (200 mg, 1.07 mmol) in DMSO (15 mL), CuSO_4_ 5H_2_O (34 mg, 0.139 mmol), sodium ascorbate (106 mg, 0.534 mmol), and 1,4-dimethyl-1-(prop-2-yn-1-yl)piperazin-1-ium (**4a**) (818 mg, 5.34 mmol). Yield: 88%. ^1^H NMR (400 MHz, D_2_O): *δ* 2.08 (N-COCH_3_), 2.44 (*N*-CH_3_) 2.90–3.04 [(H6′), (H6), Pip-3,5-CH_2_] 3.16 quaternary-*N*(CH_3_), 3.54–3.60 [(H5), quaternary-Pip-2,6-CH_2_] 3.78 (H4), 4.42 (H3), 4.57 (H2), 4.88 (triazole CH_2_ merged with D_2_O peak), 5.16 (H1), 8.59 (triazole CH).

Procedure for chitotriazolan derivative (**5**): Chitosan azide (200 mg, 1.07 mmol) in DMSO (15 mL), CuSO_4_ 5H_2_O (34 mg, 0.139 mmol), sodium ascorbate (106 mg, 0.534 mmol), and 1-(prop-2-yn-1-yl)pyridin-1-ium (**5a**) (631 mg, 5.34 mmol). Yield: 94%. ^1^H NMR (400 MHz, D_2_O): *δ* 2.08 (N-COCH_3_), 2.59 (H6′), 3.88 (H6), 3.38 (H5), 3.69 (H4), 4.38 (H3), 4.51 (H2), 5.02 [(H1) merged with D_2_O peak)], 6.06 (triazole CH_2_), 8.15 [pyridine (C3-H and C5-H)], 8.49 (triazole CH), 8.63 [pyridine (C4-H)], 9.05 [pyridine (C2-H and C6-H)].

Procedure for chitotriazolan derivative (**6**): Chitosan azide (200 mg, 1.07 mmol) in DMSO (15 mL), CuSO_4_ 5H_2_O (34 mg, 0.139 mmol), sodium ascorbate (106 mg, 0.534 mmol), and 1-methyl-3-(prop-2-yn-1-yl)-1H-imidazol-3-ium (**6a**) (646 mg, 5.34 mmol). Yield: 84%. ^1^H NMR (400 MHz, D_2_O): *δ* 2.08 (N-COCH_3_), 2.67 (H6′), 2.94 (H6), 3.44 (H5), 3.71 (H4), 3.90 (imidazole N-CH_3_), 4.42–4.56 [(H3), (H2)], 5.10 (H1), 5.64 (triazole CH_2_), 7.49–7.58 [imidazole (C1-H, C4-H, C5-H)], 8.41 (triazole CH).

Procedure for chitotriazolan derivative (**7**): Chitosan azide (200 mg, 1.07 mmol) in DMSO (15 mL), CuSO_4_ 5H_2_O (34 mg, 0.139 mmol), sodium ascorbate (105 mg, 0.534 mmol), and 1-methyl-4-(prop-2-yn-1-yl) piperazine (**7a**) (738 mg, 5.34 mmol). Yield: 75%. ^1^H NMR (400 MHz, D_2_O): *δ* 2.08 (N-COCH_3_), 2.79–3.12 [(H6′), (H6), *N*(CH_3_)], 3.34–3.94 [(Pip-2,3,5,6-CH_2_) (H5), (H4)], 4.39 (H3), 4.56 (H2), 4.75 (triazole CH_2_), 5.11 (H1), 8.51 (triazole CH).

Procedure for chitotriazolan derivative (**8**): Chitosan azide (150 mg, 0.802 mmol) in DMSO (10 mL), CuSO_4_ 5H_2_O (26 mg, 0.104 mmol), sodium ascorbate (79 mg, 0.401 mmol), and 1-(prop-2-yn-1-yl) piperazine (**8a**) (497 mg, 4.010 mmol). Yield: 60%. ^1^H NMR (400 MHz, D_2_O): *δ* 2.08 (N-COCH_3_), 2.71–3.28 [(H6′), (H6), (Pip-2,3,5,6-CH_2_)], 3.47–3.93 [(H5), (H4), (triazole CH_2_)], 4.33–4.58 [(H3), (H2)], 5.12 (H1), 8.59 (triazole CH).

Procedure for chitotriazolan derivative (**9**): Chitosan azide (200 mg, 1.07 mmol) in DMSO (15 mL), CuSO_4_ 5H_2_O (34 mg, 0.139 mmol), sodium ascorbate (106 mg, 0.534 mmol), and 2,2′-(prop-2-yn-1-ylazanediyl)bis(ethan-1-ol) (**9a**) (764 mg, 5.34 mmol). Yield: 61%. Due to a solubility issue, it could not be confirmed by proton NMR, so it was confirmed by IR spectroscopy.

### 2.3. Characterization

#### 2.3.1. ^1^H NMR and FTIR Spectra

The chitotriazolan derivatives were subjected to characterization using 1H NMR spectroscopy. The ^1^H NMR spectra were acquired using a Bruker Avance 400 spectrophotometer operating (Bruker, Rheinstetten, Germany) at a frequency of 400 MHz. NMR samples were prepared in CDCl_3_ and D_2_O solvents at concentrations ranging from 7 to 15 mg/mL. In all proton NMR spectra, the *N*-acetyl peak at 2.08 ppm served as an internal reference. Chitosan and derivatives **1** and **6** were analyzed for their ^1^H NMR and COSY spectra, which were recorded at 343 K using a Bruker Avance 400 spectrophotometer operating at a frequency of 400 MHz. The FT-IR spectra of both chitosan and the chitotriazolan derivatives were measured using a Thermo Scientific™ Nicolet™ iZ10 FTIR spectrometer (Thermo Fisher Scientific, Hvidovre, Denmark). The measurements were performed in the wavelength range of 500 to 4000 cm^−1^. The spectra were obtained by averaging 64 scans, and the resolution was set to 16 cm^−1^. A few milligrams of each material was used for the IR spectra, and all compounds were measured against a blank background.

#### 2.3.2. Gel Permeation Chromatography

Average molecular weight (Mw) determination was carried out using gel permeation chromatography (GPC) [37]. GPC measurements were taken using equipment from the Polymer Standards Service (PSS) (GmbH, Mainz, Germany): the Agilent 1260 Infinity II GPC/SEC System (Agilent Technologies GmbH, Amtsgericht Mainz, Germany), the Agilent 1260 Infinity II Isocratic Pump, the Agilent 1260 Infinity II GPC/SEC Column Thermostat for accurate temperature control and Agilent 1260 Infinity II autosampler (Agilent Technologies GmbH, Amtsgericht Mainz, Germany), the Agilent 1260 Infinity II Refractive Index Detector (Agilent Technologies GmbH, Amtsgericht Mainz, Germany), and PSS’s ETA-2010 viscometer and MALLS detector (PPC SLD 7100). WINGPC Unity 7.4 software (PSS GmbH, Mainz, Germany) was used for data collection and processing. A series of three columns [PSS Novema 10 µ guard (50 × 8 mm), PSS Novema 10 µ 30 Å (150 × 8 mm), and PSS Novema 10 µ 1000 Å (300 × 8 mm)] (PSS GmbH, Mainz, Germany) were used in the HPLC system. Ready Cal-Kit Pullulan standards with M_p_ (180–708,000 Da) from PSS (GmbH, Mainz, Germany) were used for calibration. The eluent used was a 0.1 M NaCl/0.1% TFA solution. Each sample was dissolved in the same eluent as mentioned above at a 1 mg/mL concentration at 25 °C using a flow rate of 1 mL/min. Each sample had an injection volume of 100 µL, and the time between injections was 30 min.

### 2.4. Degree of Substitution

The degree of substitution (DS) for the chitotriazolan derivatives was calculated based on the integral of the proton NMR triazole and *N*-acetyl peaks. The following equation was used to calculate the DS.

(1) $$\mathrm{DA}=\int \mathrm{Ac}/\int H_{2}-H_{5}\times 5/3$$

DS for triazole CH group:

(2) DS=(∫TzCH/∫Ac)×(3×DA)

where ∫TzCH is the integral of the triazole CH peak of a chitotriazolan derivative obtained at 8.5 ppm. The *N*-acetyl peak was assigned to 2.08 ppm for all proton NMR spectroscopy, and $\int H_{2}-H_{5}$ is the integral peak of chitosan.

### 2.5. Solubility Test in Water

A solubility test is used to determine the extent to which a substance dissolves in a particular solvent, such as water. Here is a general method for conducting a solubility test in water: Weigh all derivatives out at 8 mg/mL in distilled water. If necessary, apply vortexing or stir the sample to facilitate the dissolution process. Observe the solution closely. Note any changes in appearance, such as the formation of a clear solution, suspension, or precipitate. A clear solution indicates complete solubility, while the presence of undissolved particles indicates limited solubility or insolubility.

### 2.6. Zeta Potential Analysis

The zeta potential method is a technique used to measure the electrostatic or surface charge of the materials, and the samples were prepared in MilliQ water 1.0 mg/mL at room temperature. Zeta potential was measured using Zetasizer Nano-ZS from Malvern Instruments (Malvern Instruments Ltd., Malvern, UK) and performed dynamic light scattering (DLS) techniques with a DTS1070 capillary cell.

### 2.7. In Vitro Antibacterial Assay

The minimal inhibitory concentration (MIC) was determined following the standard procedure established by the Clinical and Laboratory Standards Institute (CLSI) [58]. The antibacterial activity was assessed against four bacterial species, namely, *Staphylococcus aureus* (*S. aureus*, ATCC 29213) and *Enterococcus faecalis* (*E. faecalis* 29212) as representative Gram-positive bacteria and *Escherichia coli* (*E. coli*, ATCC 25922) and *Pseudomonas aeruginosa* (*P. aeruginosa* 27853) as representative Gram-negative bacteria. Prior to MIC testing, the bacterial strains were cultured on a blood agar medium at 37 °C for 12–18 h. Subsequently, the bacterial colonies were suspended in saline water, adjusted to a 0.5 McFarland turbidity standard, and further diluted in Mueller–Hinton broth (MHB) to attain a final concentration of 5 × 10^5^ colony-forming units (CFU)/mL in the test wells. The MIC measurements were conducted using MHB at pH 7.2. To ensure the validity of the experiment, gentamicin, a well-established antibiotic, was employed as a performance control. MHB without any chitosan derivatives or bacterial solution served as the sterility control, while MHB containing only the bacterial solution acted as the growth control. The polymer stock solution was prepared in sterile water at a concentration of 8192 µg/mL. Subsequently, 50 µg/mL of each polymer solution was added to a 96-well microtiter plate, followed by serial dilutions in MHB to obtain concentrations ranging from 8192 to 16 µg/mL. Then, 50 µL of the bacterial suspension (5 × 10^5^ CFU/mL) was added to each well. The microtiter plates were incubated at 37 °C for 20 to 24 h, and the bacterial growth was observed visually. The MIC value was determined as the lowest concentration of the antibacterial agent that completely inhibited the visible growth of microorganisms in the 96-well microtiter plate. To determine the minimum lethal concentration (MLC), 10 µL × 2 of each dilution exhibiting no visible growth was plated on an agar plate and incubated at 35 °C for 20 to 24 h. The MLC was determined as the lowest concentration at which a 99.9% reduction in viable cells was achieved.

## 3. Results and Discussion

**Scheme 1:**
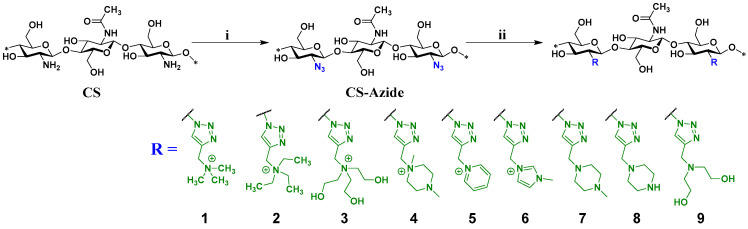
Synthetic routes of chitotriazolan derivatives. Conditions: (**i**) 0.1 M HCl solution, sodium bicarbonate, imidazole sulfonyl azide HCl salt, CuSO_4_ 5H_2_O, water, methanol, RT; (**ii**) CuSO_4_ 5H_2_O, sodium ascorbate, various alkynes, DMSO, 50 °C.

The objective of this study was to synthesize water-soluble chitotriazolan derivatives containing quaternary tetra-alkyl ammonium groups, aromatic alkyl ammonium groups, and basic protonable trialkyl amines using the CuAAC (copper-catalyzed azide–alkyne cycloaddition) method. The synthesized chitotriazolan derivatives (**1**, **2**, **4**, **5**, 7, **8**) included quaternized groups such as *N*,*N*,*N*-trimethyl and triethyl ammonium, as well as quaternary dialkyl piperazine and aromatic pyridinium groups, which have previously been found in antimicrobial *N*-alkyl and *N*-acyl chitosan derivatives [59,60,61,62] (Scheme 1). Previous research also investigated *N*-acyl chitosan derivatives with protonable piperazine and *N*-methyl piperazine groups [63]. In addition to these derivatives, we synthesized chitotriazolans with more hydrophilic trihydroxyethyl ammonium groups, *N*-methyl imidazolium groups, and basic protonable dihydroxyethyl chitotriazolan groups (derivatives **3**, **6**, and **9**). The choice of alkynes, triethylamine, and *N*,*N*,*N*-triethanolamine was similar to the *N*,*N*,*N*-trimethylamine and quaternized structure [59,63]. To confirm the conversion of azide from chitosan, we performed FT-IR spectroscopy, which showed a peak at 2109 cm^−1^, indicating the presence of azide.

**Figure 1:**
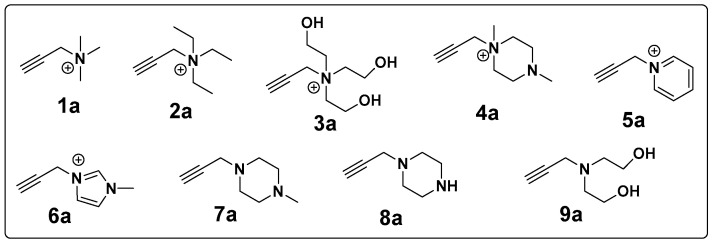
Different alkyne moieties.

Various alkynes were prepared by reacting propargyl bromide with a trimethylamine solution, triethylamine, triethanolamine, *N*,*N*-dimethylpiperazine, *N*-methyl imidazole, pyridine, *N*-methylpiperazine, piperazine, and *N*,*N*-diethanolamine (Figure 1). The alkynes’ proton NMR results are shown in the supporting information. These alkynes were conjugated to the chitosan backbone structure through the CuAAC reaction, resulting in the synthesis of chitotriazolan derivatives. The conversion of triazole and quaternized functional groups was confirmed by proton NMR and the disappearance of the azide peak in the FT-IR spectrum. The choice of alkynes, triethylamine, and *N*,*N*,*N*-triethanolamine was similar to the *N*,*N*,*N*-trimethylamine and quaternized structure [59,63].

### 3.1. Characterization by IR and NMR Spectroscopy

**Figure 2:**
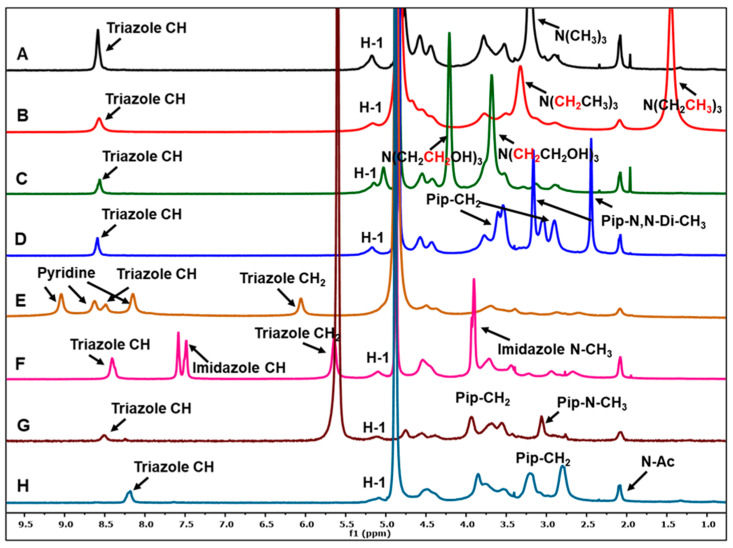
Proton NMR spectra of all chitotriazolan derivatives: derivative **1** (**A**), derivative **2** (**B**), derivative **3** (**C**), derivative **4** (**D**), derivative **5** (**E**), derivative **6** (**F**), derivative **7** (**G**), derivative **8** (**H**).

The chitotriazolan derivatives were characterized using IR and NMR spectroscopy (Figure 2). The FT-IR spectrum showed the presence of the azide moiety in chitosan azide, indicated by the peak at 2109 cm^−1^. This peak disappeared when the C-2 azido group was transformed into 1,2,3-triazoles. Additionally, the proton NMR spectrum exhibited a new peak in the range of 8.19 to 8.59 ppm, corresponding to the C-H alkene protons of the 1,2,3-triazole derivatives. The reference protons for all derivatives were observed at 2.08 ppm, representing the chitosan *N*-acetyl peak.

The *N*-(CH_3_)_3_ proton peak was observed at 3.21 ppm for derivative **1** (Figure 2A), consistent with our previous report. For derivative **2**, the *N*-(CH_2_CH_3_)_3_ proton peaks were observed at 1.45 ppm and 3.33 ppm, corresponding to the ethyl CH_3_ and ethyl CH_2_ protons, respectively (Figure 2B). The triethanolammonium *N*-(CH_2_CH_2_OH)_3_ proton peaks were obtained at 3.68 ppm and 4.21 ppm (Figure 2C). In the proton spectra of derivative **4**, the peaks corresponding to Pip-*N*-CH_3_, Pip-3,5-CH_2_, quaternary-*N*(CH_3_), and quaternary-Pip-2,6-CH_2_ were observed at 2.44 ppm, 2.90–3.04 ppm, 3.16 ppm, and 3.54–3.60 ppm, respectively. Figure 2E represents the proton spectrum of pyridine derivative **5**, which showed a new signal attributed to the five aromatic protons of pyridine, and the triazole-CH_2_ protons shifted slightly upfield to 6.06 ppm. In the proton spectrum of imidazole click derivative **6** (Figure 2F), signals for the three alkene protons of imidazole (C1-H, C4-H, C5-H) were observed at 7.49–7.58 ppm. These spectra further confirmed the appearance of the *N*-methyl peak at 3.90 ppm and the upfield shift of the triazole CH_2_ protons to 5.64 ppm. The proton peaks for N-methyl piperazine derivative **7** were observed at 3.34–3.94 ppm for (Pip-2,3,5,6-CH_2_) and 3.06 ppm for *N*-methyl protons. In the case of derivative **8**, a signal for piperazine was observed in the range of 2.71–3.28 ppm (Pip-2,3,5,6-CH_2_), merging with chitosan’s H-6 and H-6′ protons.

**Figure 3:**
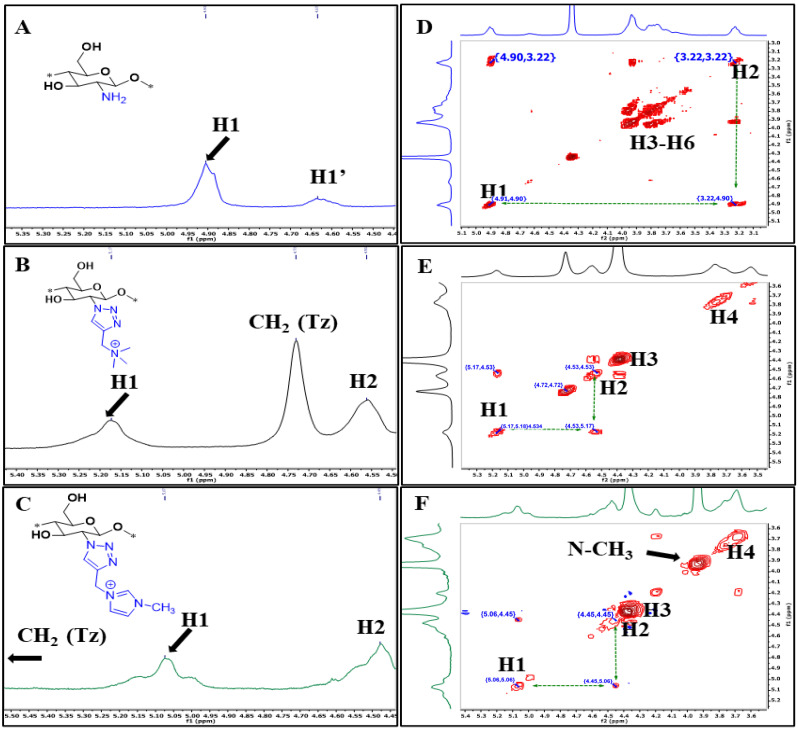
Proton NMR and COSY NMR spectra were measured at 343 K. Proton NMR for chitosan (**A**), derivative **1** (**B**), and derivative **6** (**C**); COSY spectra for chitosan (**D**), derivative **1** (**E**), and derivative **6** (**F**). The full spectra are available in the supporting information.

Chitosan and chitotriazolan derivatives **1** and **6** were further analyzed by proton NMR and COSY spectra obtained at 343 K. The increased temperature caused an upfield shift of the HDO/water peak, and thus, it was possible to observe the H1 peaks in the anomer region (see Figure 3). The H1 peak for chitosan was observed at 4.90 ppm, whereas this peak was shifted to 5.18 and 5.06 ppm in chitotriazolan derivatives **1** and **6,** respectively. In the latter spectrum, there is no residual peak at 4.90 ppm, confirming the full conversion of 2-NH_2_ to a triozole moiety. In the COSY spectra, Figure 3D,E indicate that the H2 peak for chitosan is observed at 3.2 ppm, while the H1 and H2 cross-peaks are observed at 4.90 and 3.22 ppm, respectively. In contrast, the H2 peaks of chitotriazolan derivatives **1** and **6** were shifted downfield to 4.53 and 4.45 ppm, respectively. Further supporting that conversion to triazole was complete. The molecular weights (MWs) of chitotrizolan derivatives were determined by gel permeation chromatography, and the MWs are shown in Table 1. There is an MW reduction in the polymer chain due to azide conversion and the click reaction. The polymer surface charges were measured by the zeta potential, and the values range between 15.6 and 52.2 mV (Table 1). It has been confirmed that quaternary derivatives have a higher positive charge compared to non-quaternary derivatives. The chromatogram is provided in the supporting information.

### 3.2. Solubility Analysis in Water

**Figure 4:**
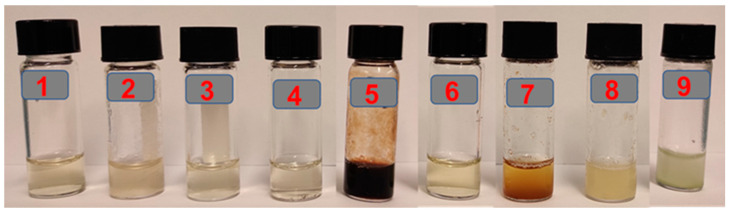
Solubility test for all chitotriazolan derivatives **1**–**9**.

The water solubilities of the chitotriazolan derivatives in distilled water at 25 °C were determined (Figure 4). Chitosan was soluble in acidic solutions at room temperature, while chitosan azide was soluble only in DMSO/DMF at 50 °C. However, all chitotriazolan derivatives demonstrated good water solubility at room temperature. At a concentration of 8 mg/mL, the solubilities of all derivatives were measured. Derivatives containing quaternary or cationic chitosan triazole groups exhibited the clearest visible solubility. Other derivatives that did not contain quaternary groups showed somewhat reduced solubility. Specifically, derivatives **1**–**6** were highly soluble in water, resulting in clear solutions, while derivatives **7** and **8** were moderately soluble, forming cloudy solutions. Derivative **9** had the least solubility, with some undissolved particles present.

The degree of substitution (DS) could not easily be determined from the NMR spectra due to the extensive overlap of the peaks. However, the triazole CH peak and the acetyl CH_3_ peak at 8.4 to 8.5 ppm and 2.08 ppm, respectively, were fully resolved from other peaks and could be used to estimate the DS. This was based on the assumption that the conversion with chitotriazolan did not change the DA (from 0.18) (Table 1). This analysis confirmed extensive conversion, and the estimated DS was 0.44 to 0.77. However, this was certainly an underestimate, as the further NMR analysis of **1** and **6** at 343 K (shown in Figure 3) showed that there was full conversion, so the “true” DS should be around 0.82. The isolated yield, calculated based on one monomer unit, was excellent for most derivatives, ranging from 80% to 94%. However, derivatives **8** and **9** had yields of 60% and 61%, respectively.

### 3.3. Investigation of Antibacterial Activity of Chitotriazolan Derivatives

The antimicrobial properties of chitosan derivatives, particularly quaternized chitosan derivatives, have attracted the attention of researchers. In this study, quaternized and neutral chitotriazolan derivatives were evaluated for their antibacterial activity against Gram-positive and Gram-negative microorganisms, including *S. aureus*, *E. faecalis*, *E. coli*, and *P. aeruginosa*. The minimum inhibitory concentration (MIC) and minimum lethal concentration (MLC) values (µg/mL) were determined and are presented in Table 2.

The cationic quaternary chitotriazolan derivatives exhibited effective activity against all bacteria, with MIC values ranging from 256 to 8192 µg/mL. Among these derivatives, the trimethylammonium chitotriazolan derivatives showed the most potent activity against all tested bacteria. Surprisingly, derivative **1** had an MIC of 512 µg/mL against *S. aureus*, which was three times less active than reported in our previous study. Derivative **2**, which is structurally similar to derivative **1** but contains *N*,*N*,*N*-triethylammonium groups, exhibited lower activity against Gram-positive bacteria but moderate activity against Gram-negative bacteria. Piperazine derivatives **4**, **7**, and **8** were not active against any of the tested bacteria, whereas derivative **4** showed better activity. Derivatives **3**, **5**, and **6** demonstrated better activity against all bacteria, with MIC values ranging from 256 to 8192 µg/mL. Notably, derivative **6** was the most active in this series of studies, with an MIC of 256 µg/mL, except for *E. faecalis*. However, the diethanolammonium derivative **9** did not exhibit any antibacterial activity. The MLC values showed similar trends to the MIC values, except for derivatives **1**, **2**, and **8**, which showed different activity against *P. aeruginosa*.

Interestingly, a relationship was observed between the measured z-potential, as reported in Table 1, and the antimicrobial activity, as noted in Table 2. Specifically, an increase in z-potential was associated with a decrease in the MIC, showing enhanced activity. This correlation proved consistent across all tested bacteria, though it was only statistically significant for *P. aeruginosa* (see Supplementary Materials Figure S1). Therefore, it is plausible that the increased net positive charge of the polymeric particles facilitates greater interaction with and disruption of the negatively charged bacterial cell membranes. Previous publications by our group and others have suggested that the cytoplasmic membrane serves as the primary target for chitosan and its derivatives [64,65,66,67].

## 4. Conclusions

In this study, we successfully synthesized cationic quaternized chitotriazolan derivatives through the use of a CuAAC reaction. The nine derivatives were characterized using FT-IR and proton NMR spectroscopy. The estimated DS based on the triazole CH peak and the acetyl peak was between 0.44 and 0.77, but further NMR analysis of the anomer region at 343 K confirmed the full conversion of the NH_2_ groups to triazole moieties. We investigated the antibacterial activity of these derivatives against *S. aureus*, *E. faecalis*, *E. coli*, and *P. aeruginosa* and found that the cationic quaternized derivatives had stronger antibacterial activity than the non-quaternized ones. *N*-methylimidazole chitotriazolan derivatives showed the best activity at 256 µg/mL, except for *E. faecalis*. The future direction of our study involves expanding the investigation to include activity against fungi, additional bacteria, and viruses. Antibiofilm activity also represents an important area of focus. We aim to incorporate these studies into part of our broader effort to understand the structure–activity relationship and mechanisms underlying the antimicrobial properties of chitosan derivatives.

## Figures and Tables

**Table 1:** Physicochemical properties of chitotriazolan derivatives.

Chitosan Derivatives	Yield (%)	DS (Based on Triazole CH and Acetyl Peak)	MW (Da)	Polydispersity Index (D)	Zeta Potential (mV)
**1**	82	(0.74)	7.055 × 10^4^	2.30	46.4
**2**	91	(0.77)	1.046 × 10^5^	3.92	51.5
**3**	80	(0.44)	3.225 × 10^5^	3.93	47.0
**4**	88	(0.53)	1.709 × 10^5^	1.80	35.1
**5**	94	(0.73)	1.164 × 10^5^	1.23	39.5
**6**	84	(0.70)	1.330 × 10^5^	2.77	52.2
**7**	75	(0.54)	2.721 × 10^4^	1.18	16.3
**8**	60	(0.57)	1.699 × 10^4^	1.00	32.7
**9**	61	NA *	4.724 × 10^5^	1.23	15.6

* NA—not available.

**Table 2:** Antibacterial activity (MIC and MLC) of chitotriazolan derivatives.

Chitosan Derivatives	Structures	*S. aureus* µg/mL	*E. faecalis* µg/mL	*E. coli* µg/mL	*P. aeruginosa* µg/mL
**1**	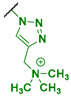	512	8192	512	1024 *
**2**	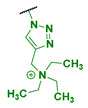	8192	8192	512	1024 *
**3**	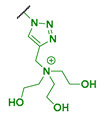	256	8192	4096	512
**4**	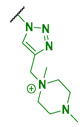	4096	8192	1024	4096
**5**	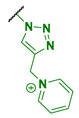	512	2048	512	1024
**6**	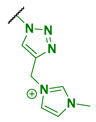	256	4096	256	256
**7**	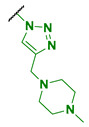	8192	8192	8192	≥8192
**8**	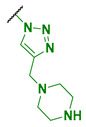	≥8192	≥8192	≥8192	8192 *
**9**	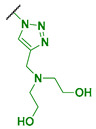	≥8192	≥8192	≥8192	≥8192

* MLC values are different from MIC values for the marked derivatives; the others are the same.

## Data Availability

The original contributions presented in the study are included in the article and Supplementary Material, further inquiries can be directed to the corresponding authors.

## Supplementary Materials

The following supporting information can be downloaded at: https://www.mdpi.com/article/10.3390/jfb15030063/s1, Figure S1: Correlation between MIC values and z-potential; Figure S2: Zeta potential chromatogram (Derivative **1**–**9**); Figure S3: Proton NMR of **1**–**8**; Figure S4: Proton NMR for Chitosan at 343 K; Figure S5: COSY NMR for Chitosan at 343 K; Figure S6: Proton NMR for Chitotriazolan derivatives **1** at 343 K; Figure S7: COSY NMR for Chitotriazolan derivatives **1** at 343 K; Figure S8: Proton NMR for Chitotriazolan derivatives **6** at 343 K; Figure S9: COSY NMR for Chitotriazolan derivatives **6** at 343 K; Figure S10: Proton NMR of **1a**–**9a**; Figure S11: IR Spectra of Chitosan Azide and Compounds **1**–**9**.
