# Supplementary material for: Water-Soluble Quaternary and Protonable Basic Chitotriazolans: Synthesis by Click Chemistry Conversion of Chitosan Azides and Investigation of Antibacterial Activity
Source: J Funct Biomater. 2024 Mar 5;15(3):63. doi: 10.3390/jfb15030063 (PMC10971457; doi:10.3390/jfb15030063)
Supplement: Supplementary file 1 [file jfb-15-00063-s001.zip › jfb-2828616-supplementary.pdf]

Journal of
Functional Biomaterials

Supplementary Materials

Water-Soluble Quaternary and Protonable Basic Chitotriazolans: Synthesis by Click Chemistry Conversion of Chitosan Azides and Investigation of Antibacterial Activity

Sankar Rathinam [1], Romano Magdadaro [1], Martha Á. Hjálmarsdóttir [2] and Már Másson [1,*]

[1] Faculty of Pharmaceutical Sciences, School of Health Sciences, University of Iceland, Hofsvallagata 53, IS-107 Reykjavík, Iceland; sankar@hi.is (S.R.); c18434796@mytudublin.ie (R.M.)
[2] Department of Biomedical Science, Faculty of Medicine, School of Health Sciences, University of Iceland, Hringbraut 31, IS-101 Reykjavík, Iceland; hjalmars@hi.is
* Correspondence: mmasson@hi.is

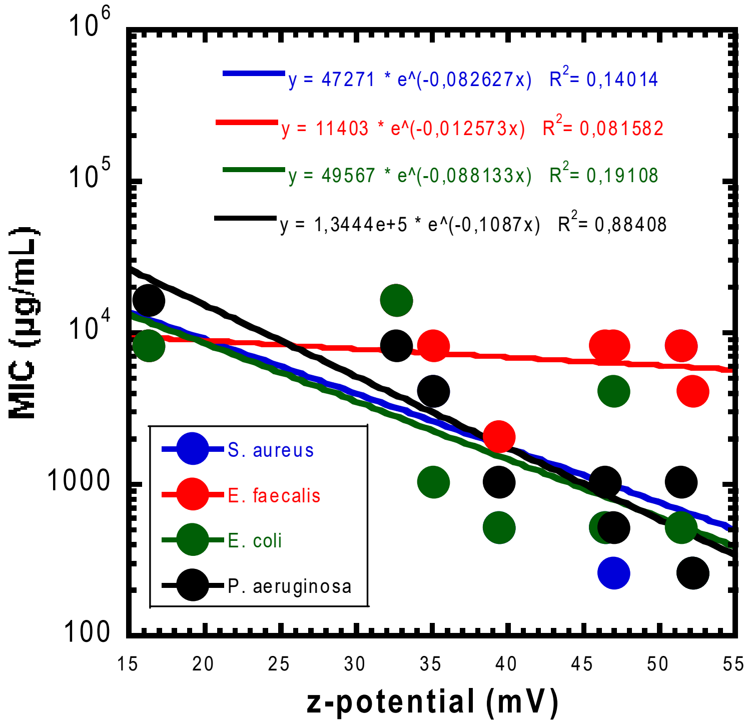

Figure S1. Correlation between MIC values and z-potential.

Derivative 1

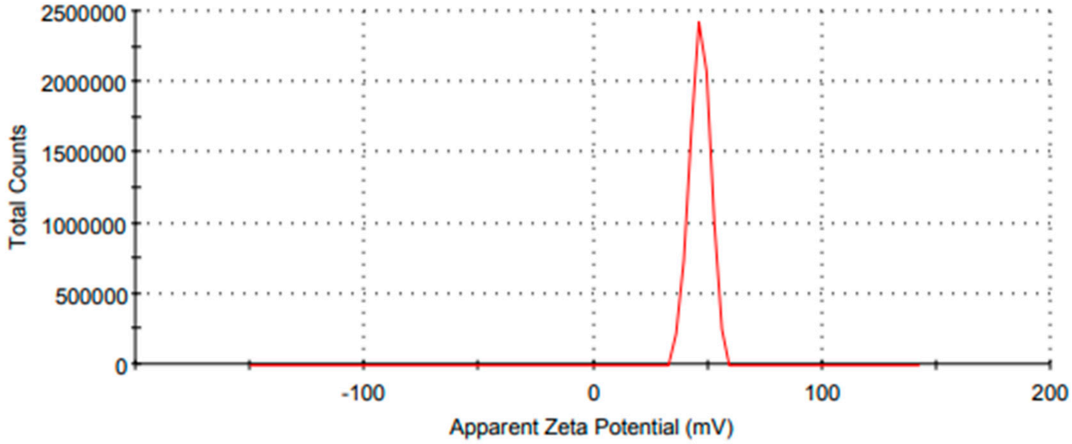

Derivative 2

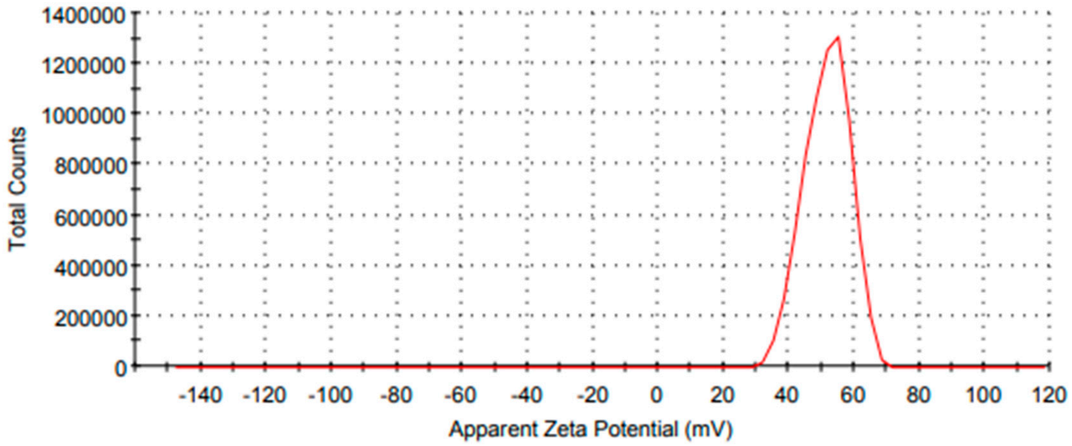

Derivative 3

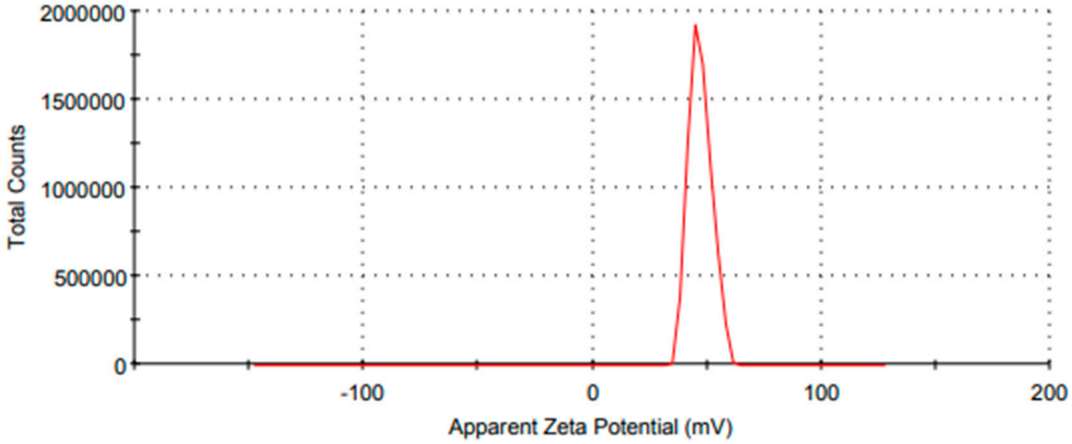

Derivative 4

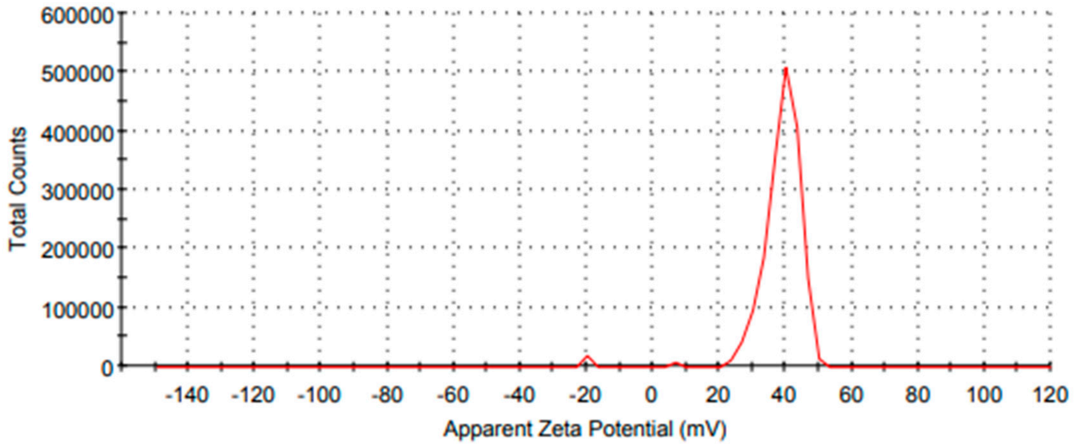

Derivative 5

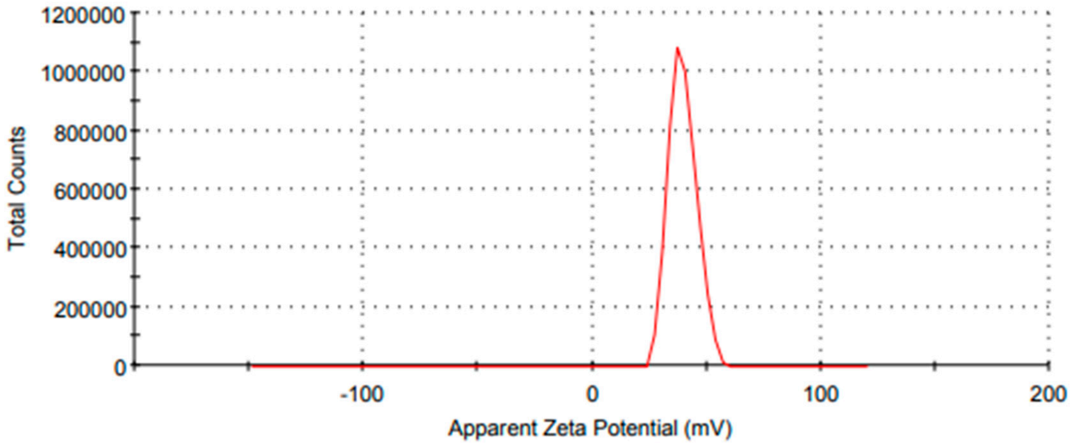

Derivative 6

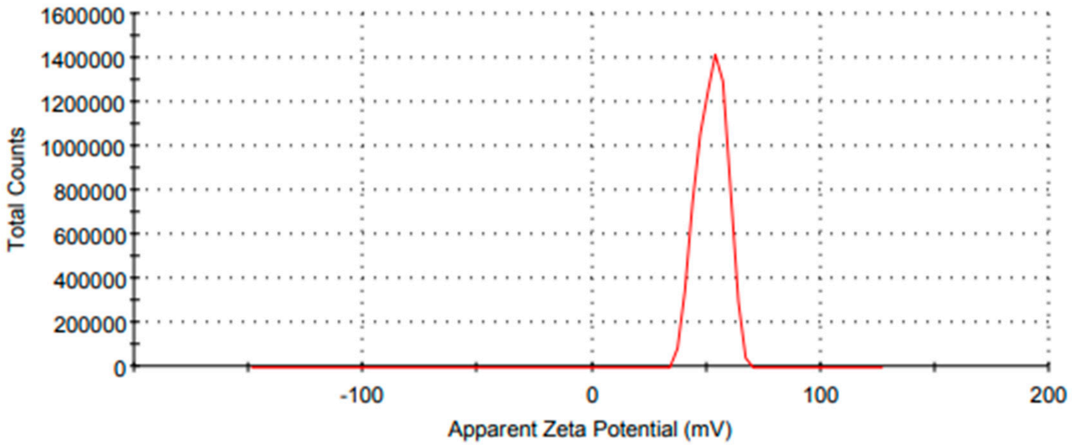

Derivative 7

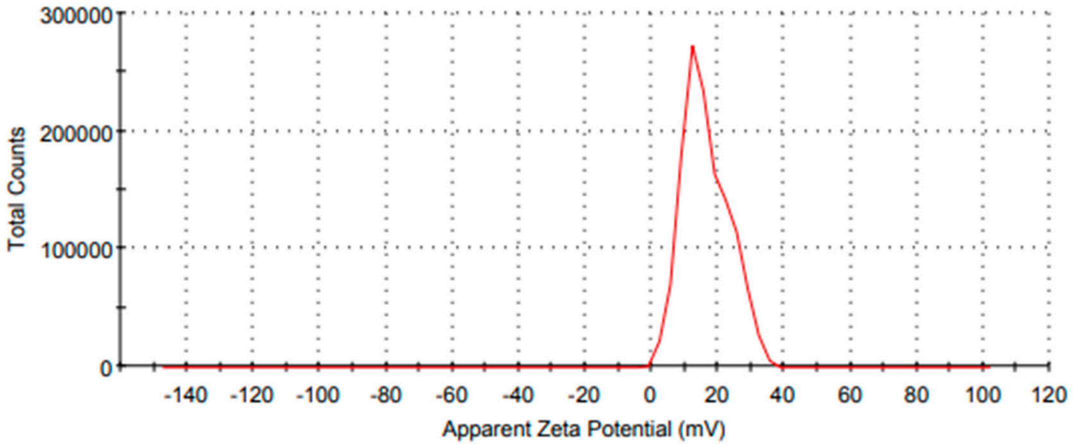

Derivative 8

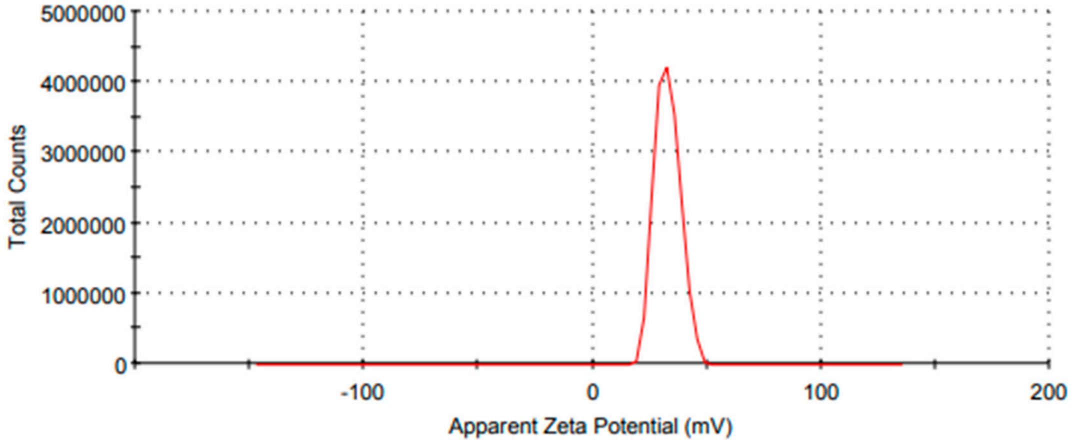

Derivative 9

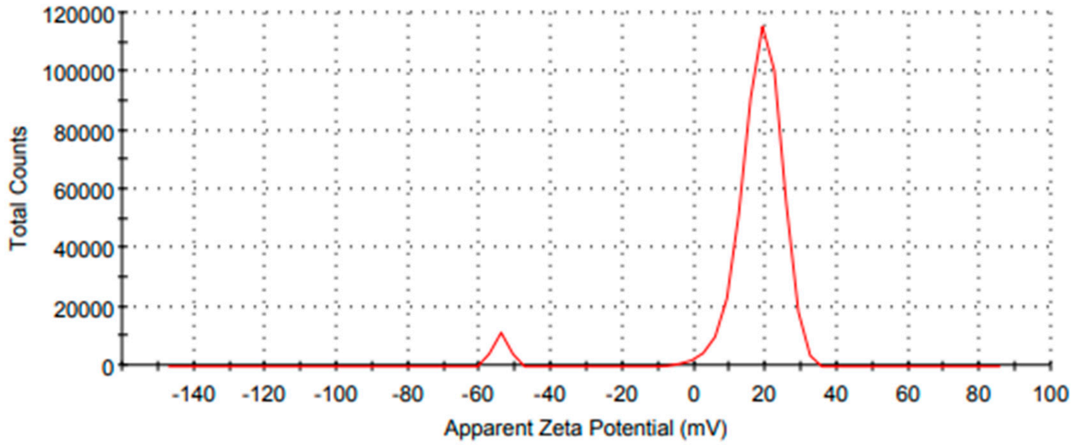

Figure S2. Zeta potential chromatogram (Derivative **1–9**).

Proton NMR of 1

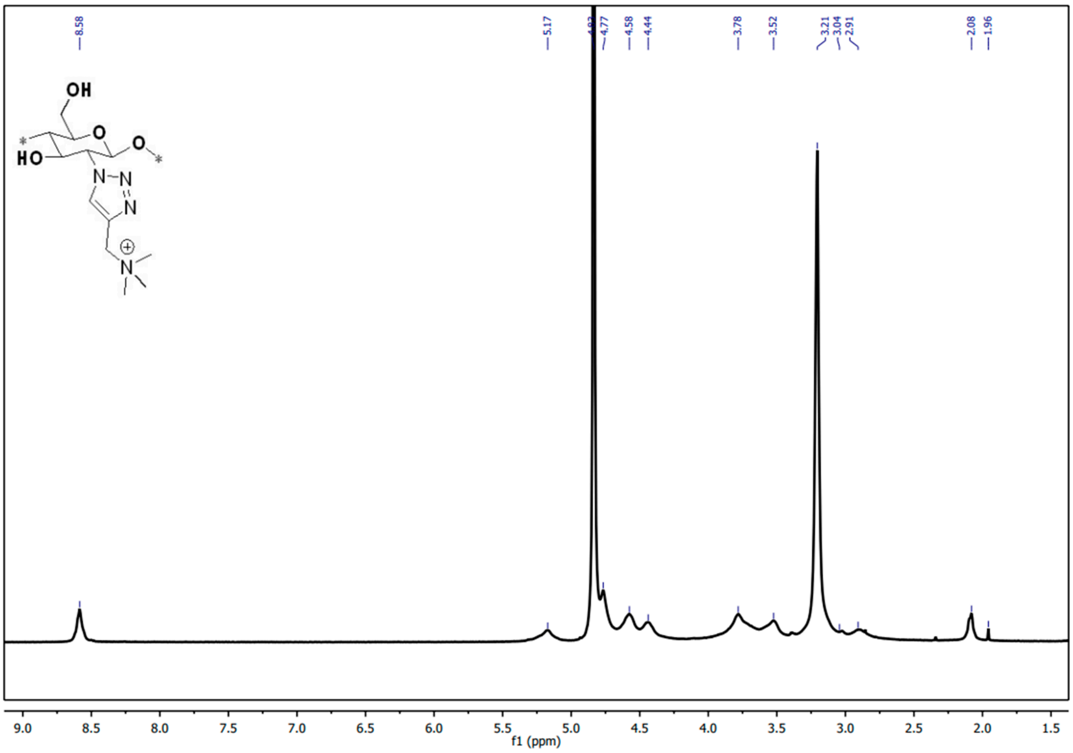

Proton NMR of 2

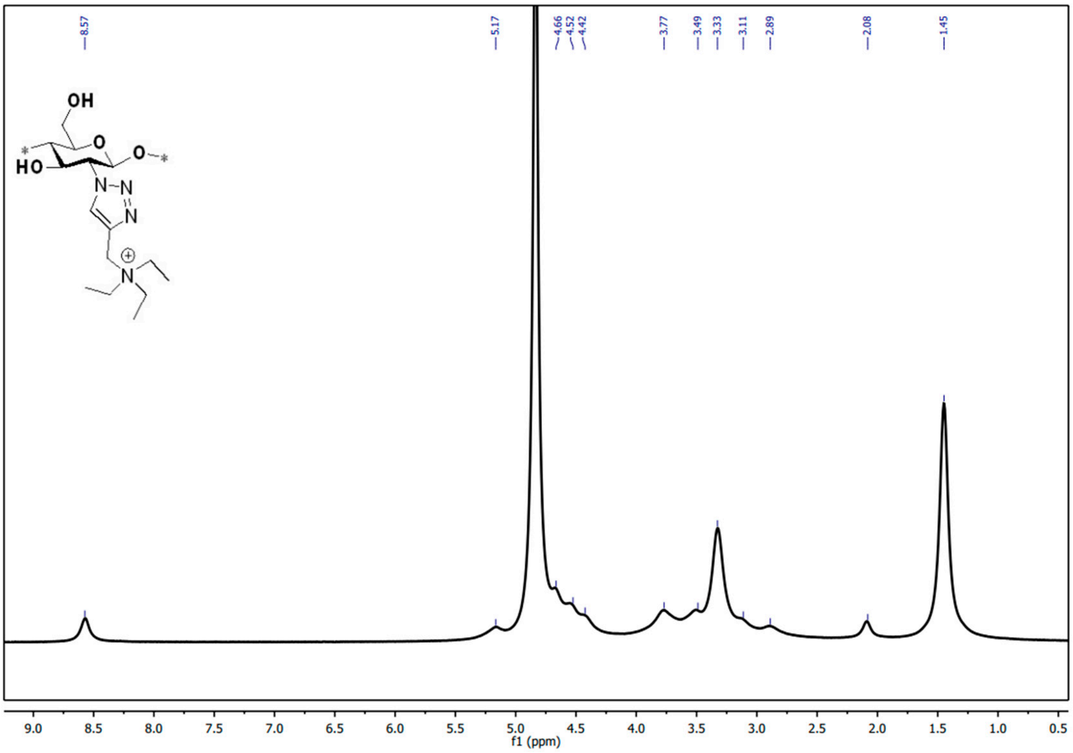

Proton NMR of 3

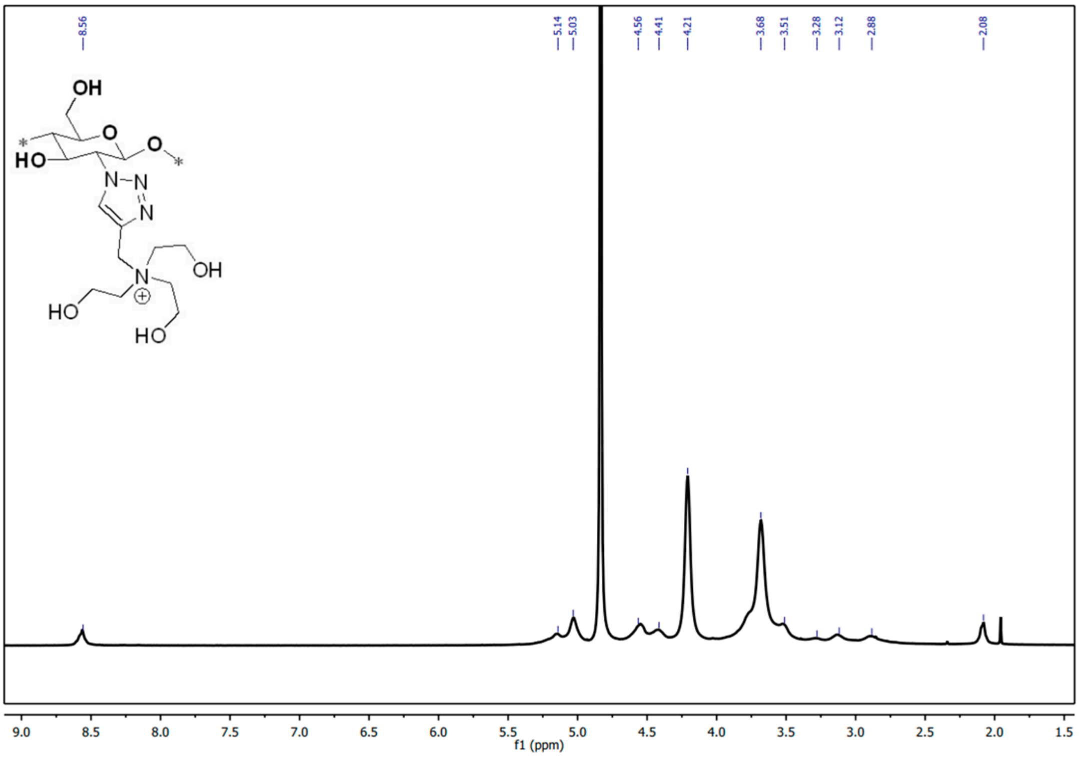

Proton NMR of 4

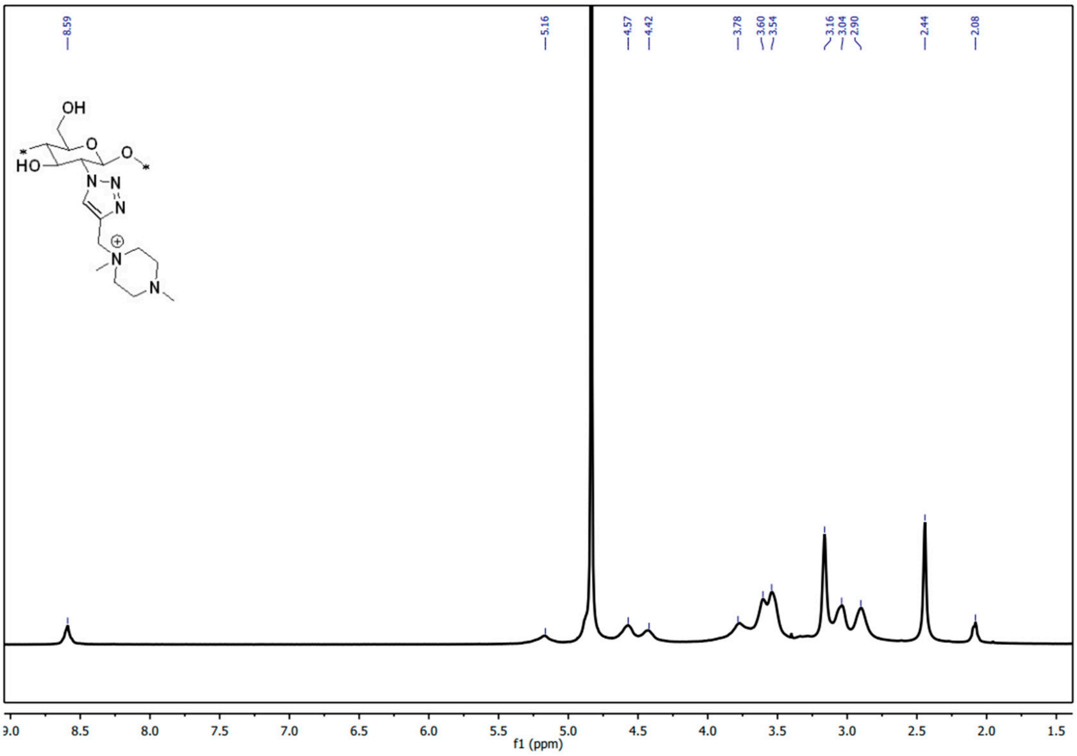

Proton NMR of 5

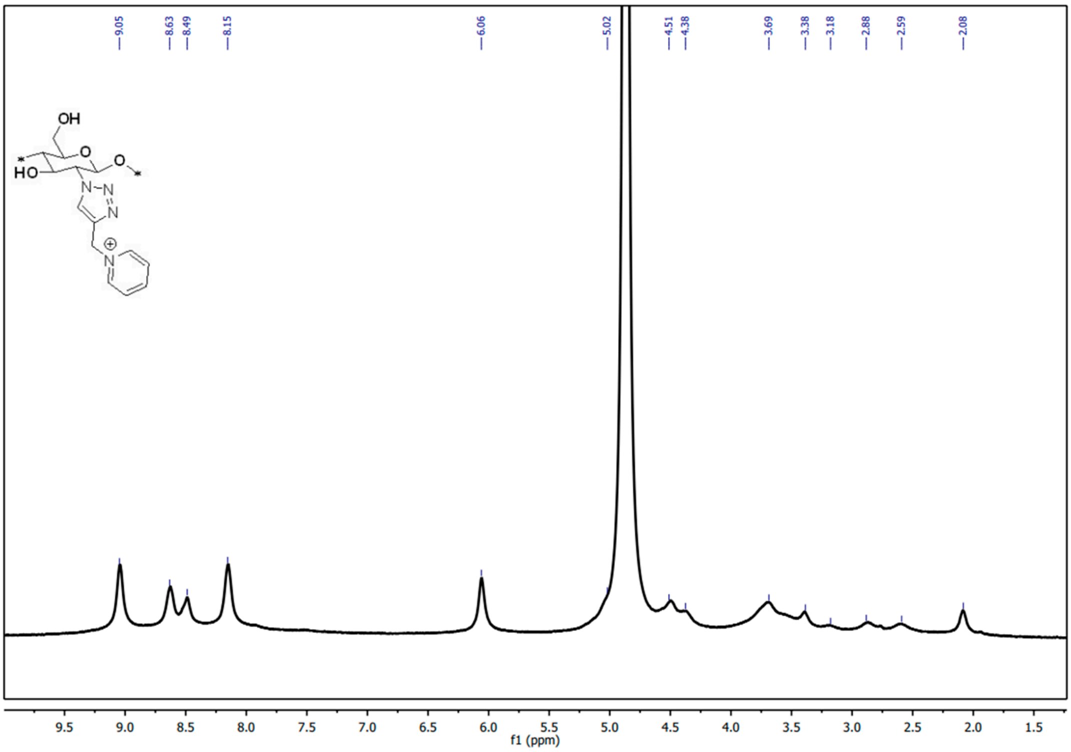

Proton NMR of 6

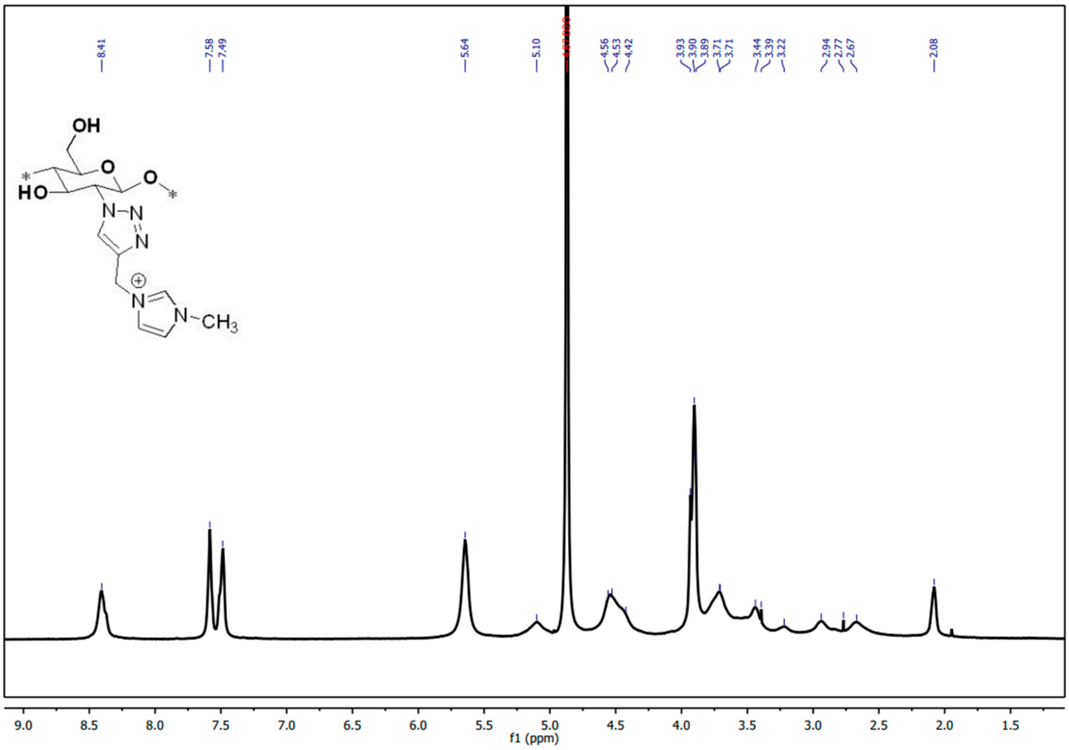

Proton NMR of 7

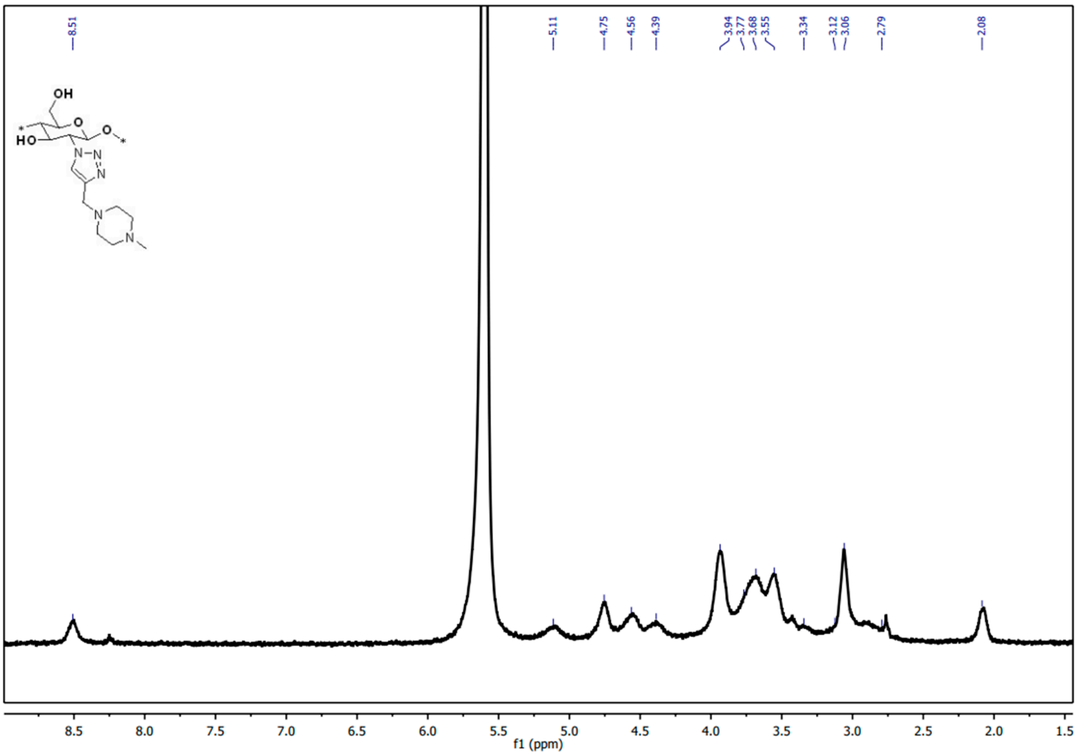

Proton NMR of 8

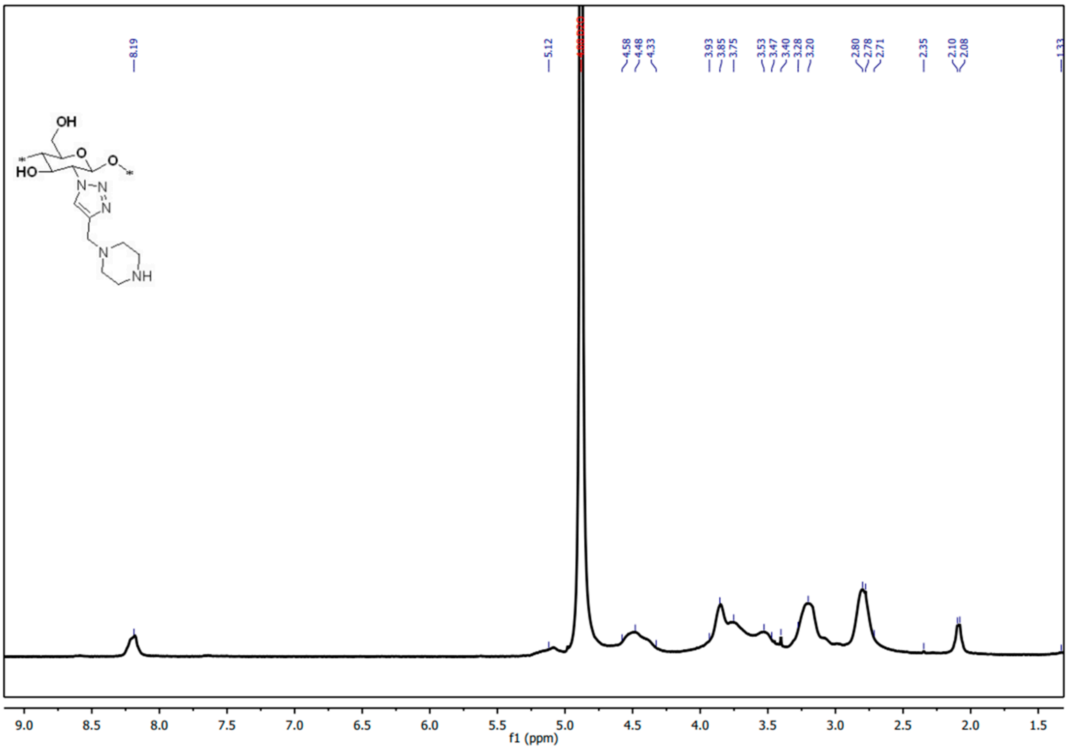

Figure S3. Proton NMR of **1–8**.

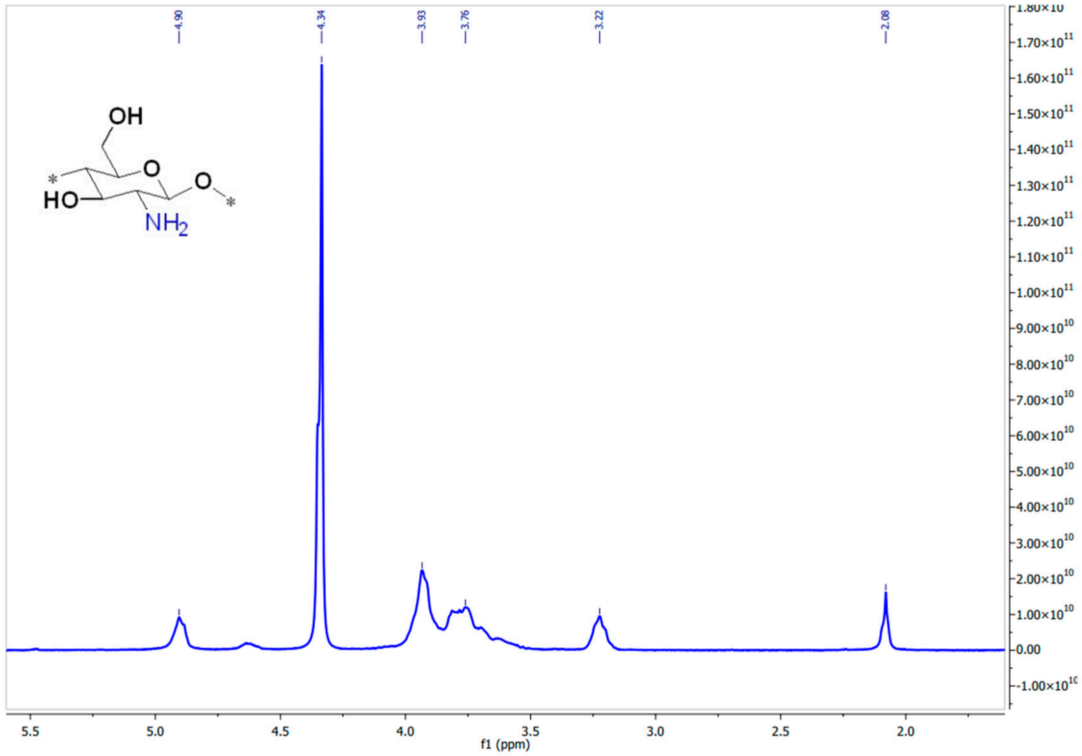

Figure S4. Proton NMR for Chitosan at 343 K.

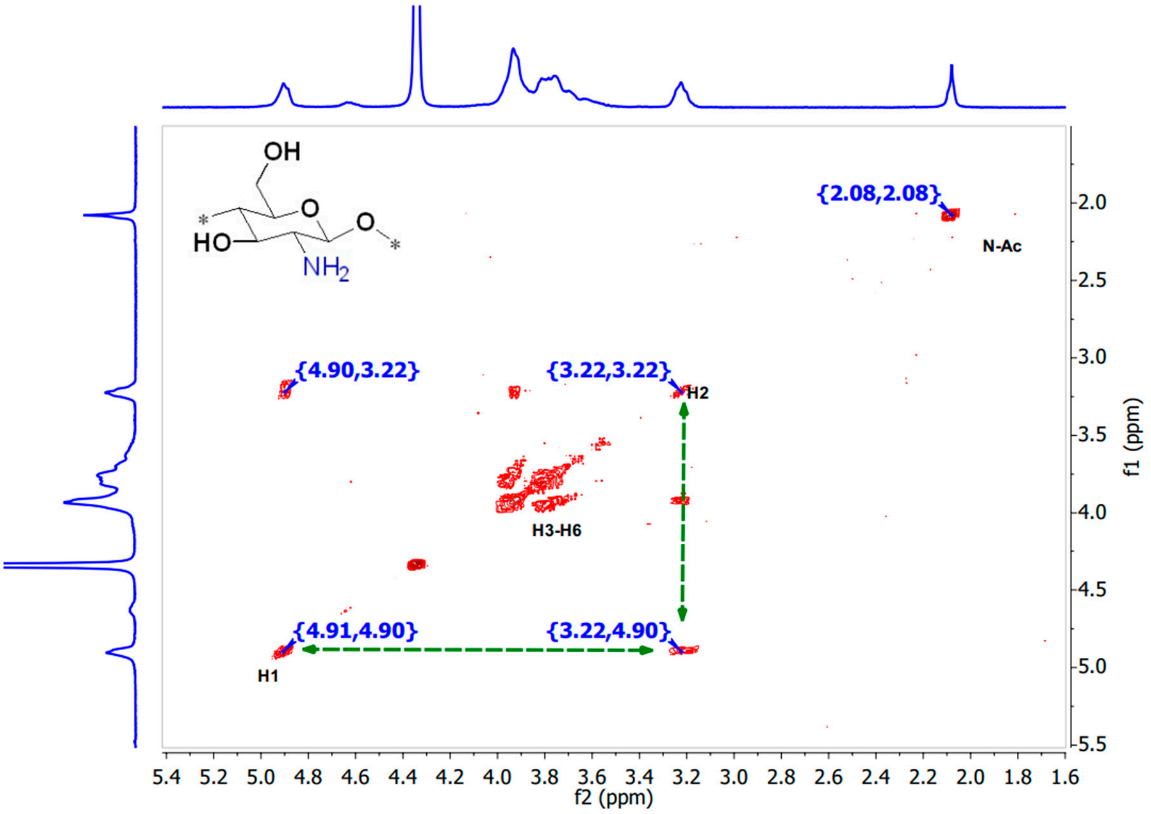

Figure S5. COSY NMR for Chitosan at 343 K.

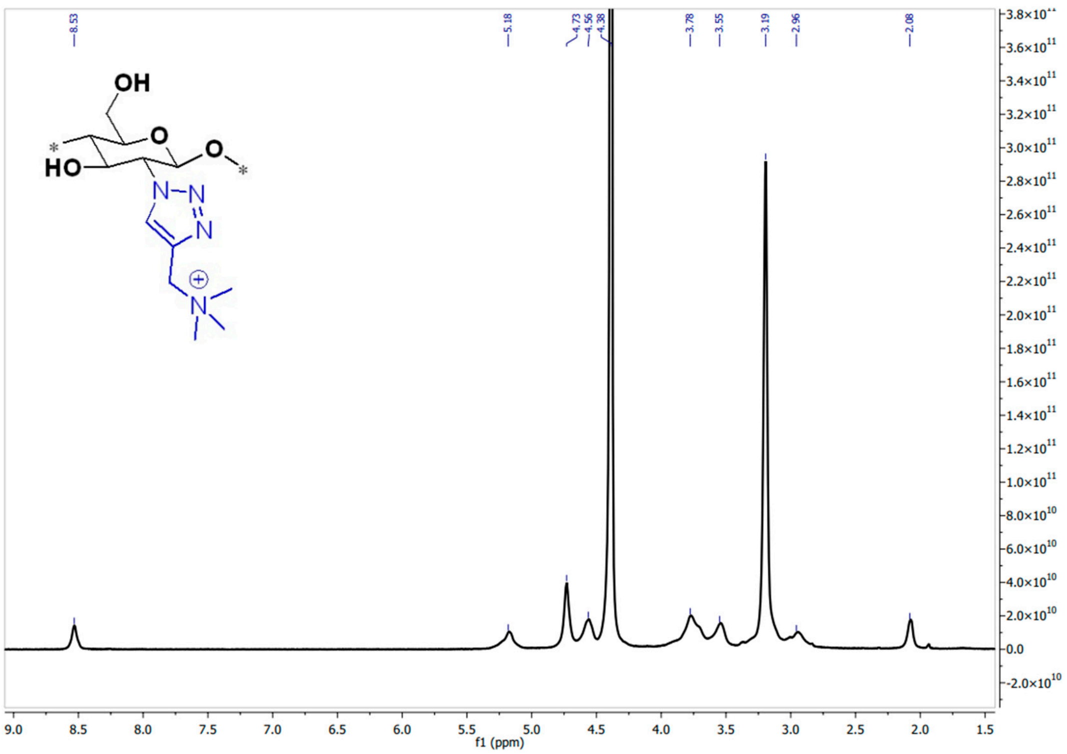

Figure S6. Proton NMR for Chitotriazolan derivatives **1** at 343 K.

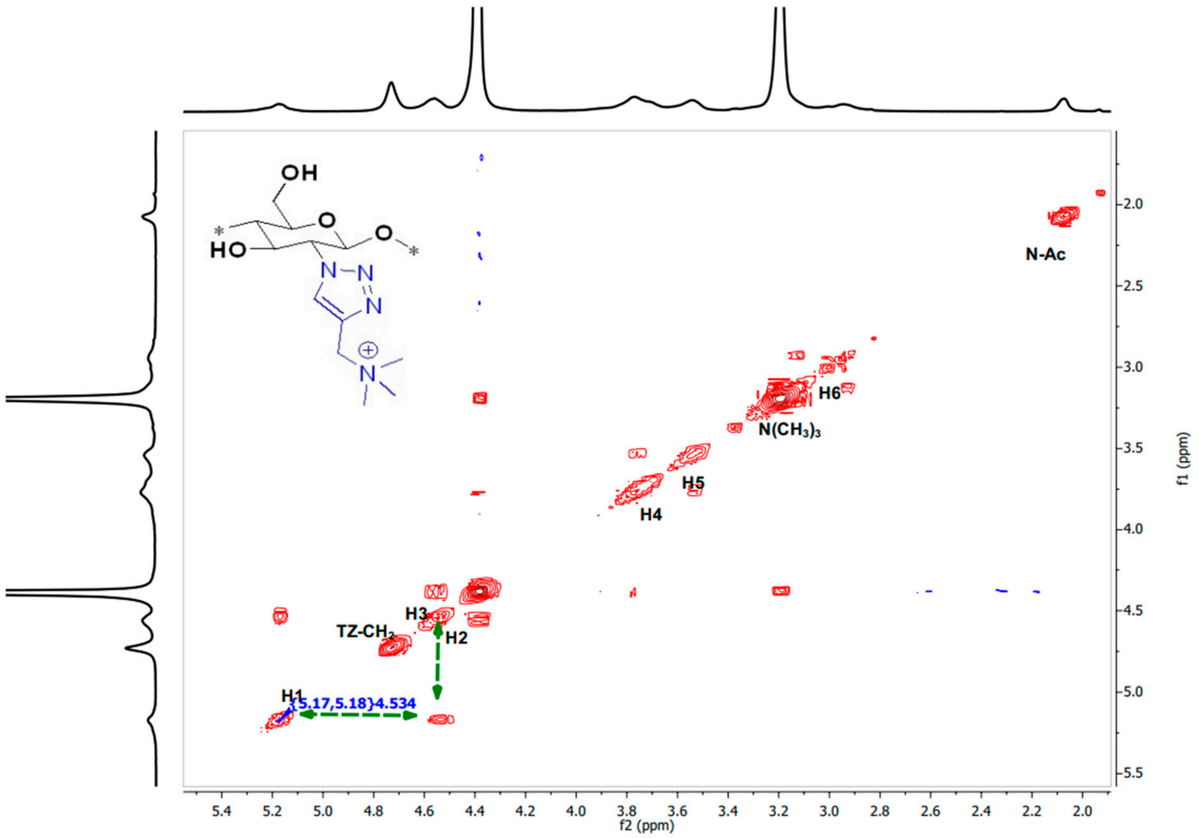

Figure S7. COSY NMR for Chitotriazolan derivatives **1** at 343 K.

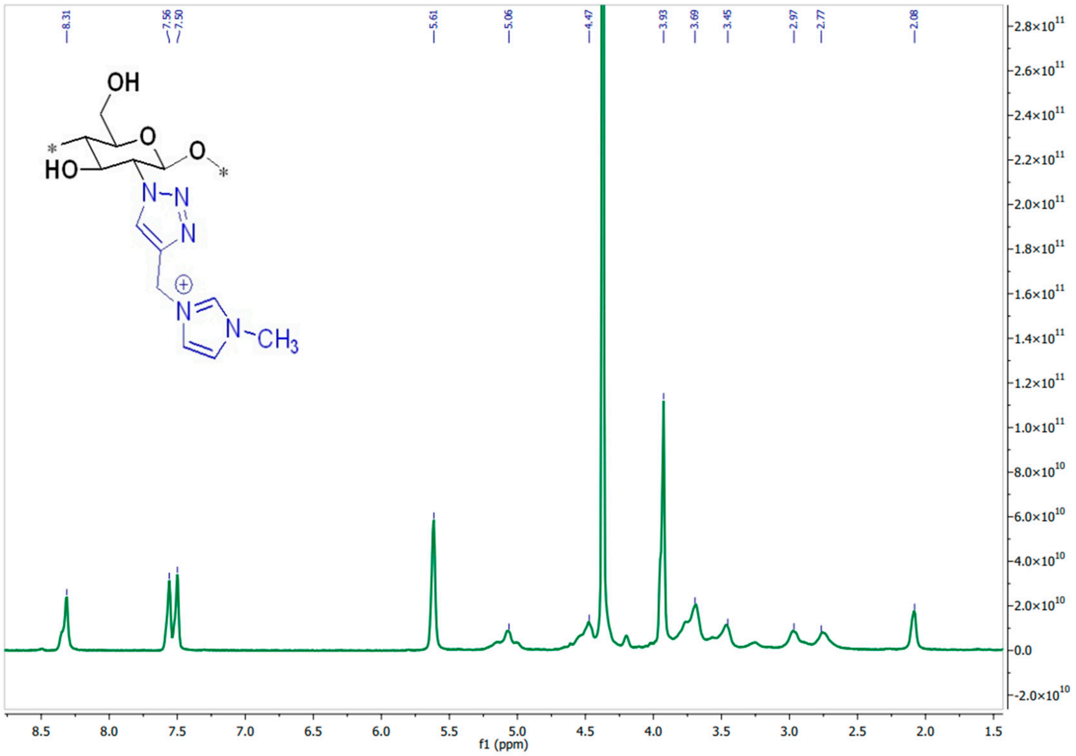

Figure S8. Proton NMR for Chitotriazolan derivatives **6** at 343 K.

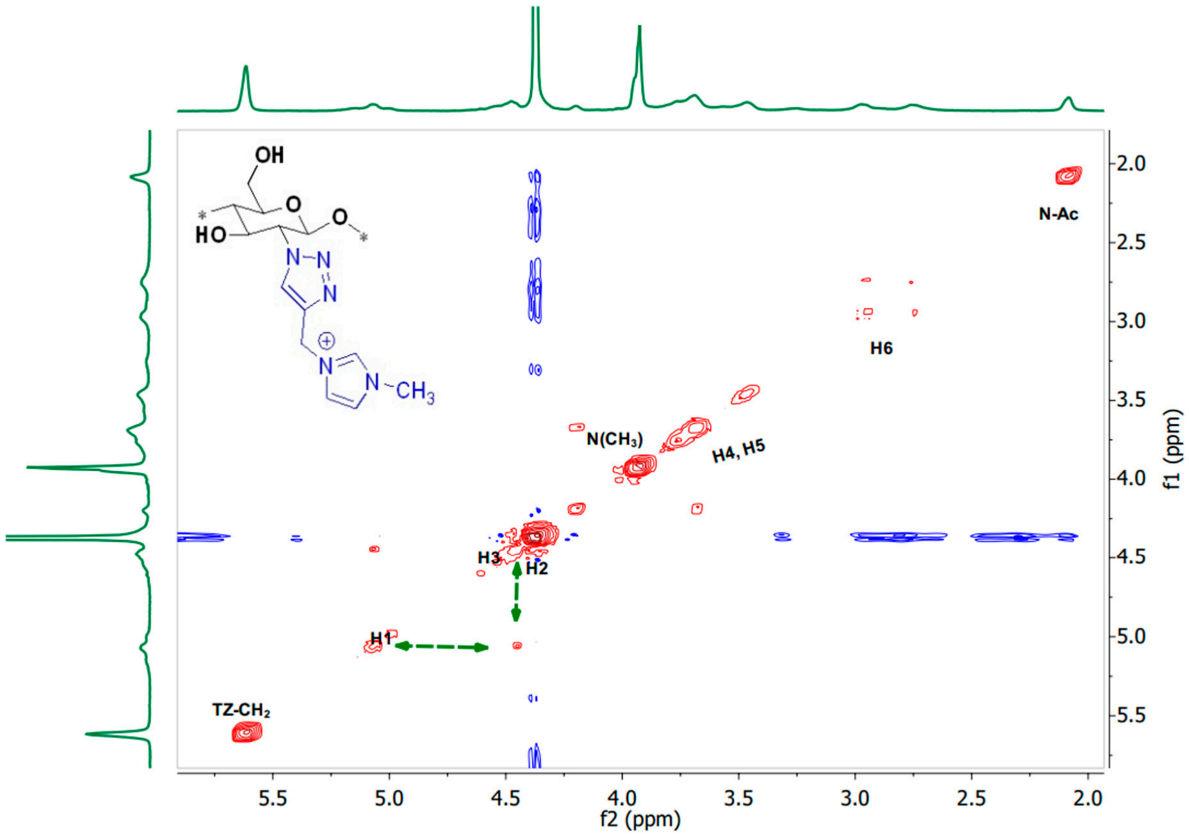

Figure S9. COSY NMR for Chitotriazolan derivatives **6** at 343 K.

Proton NMR of 1a

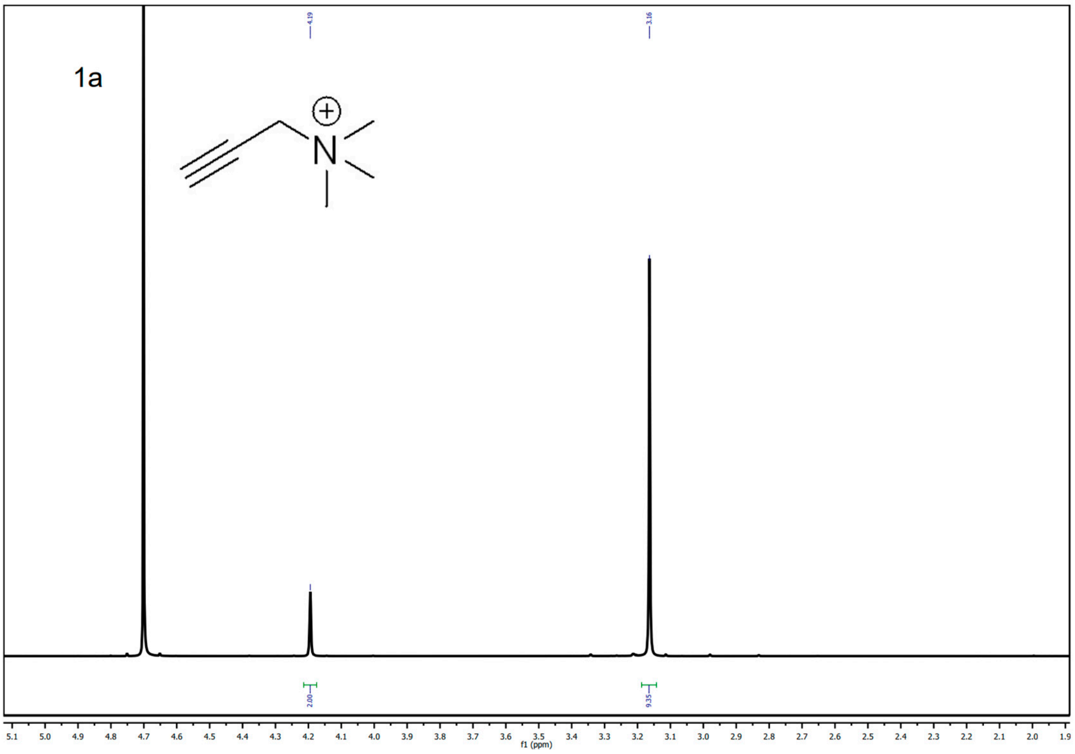

Proton NMR of 2a

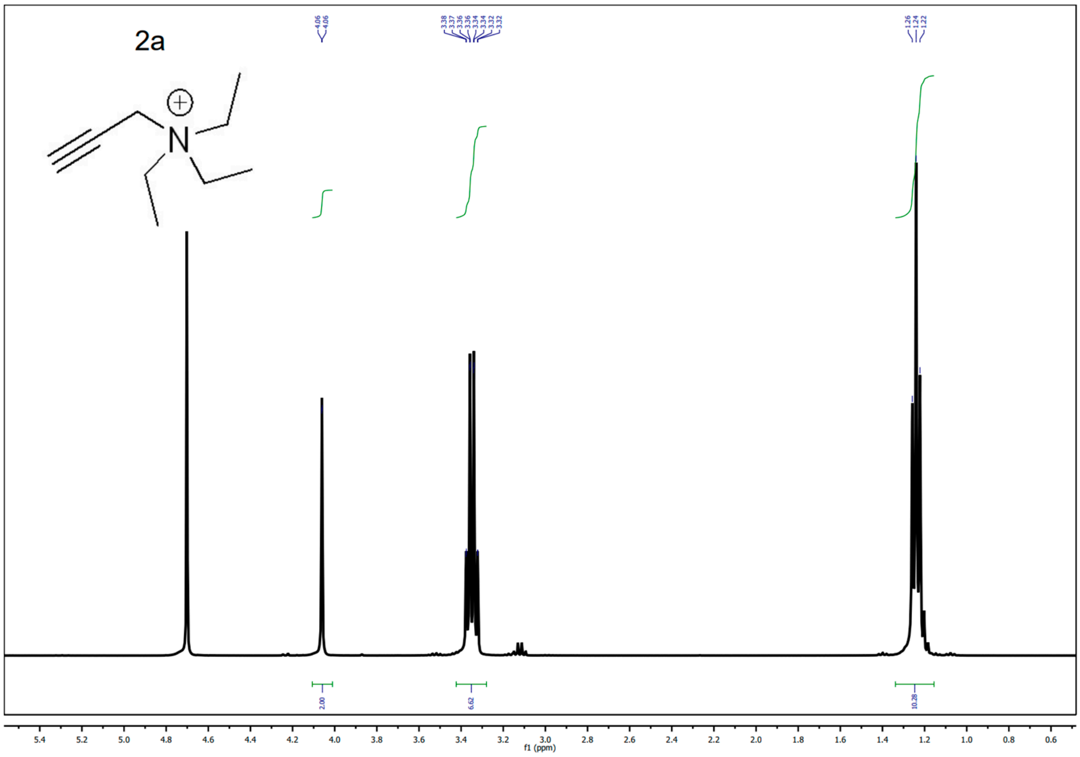

Proton NMR of 3a

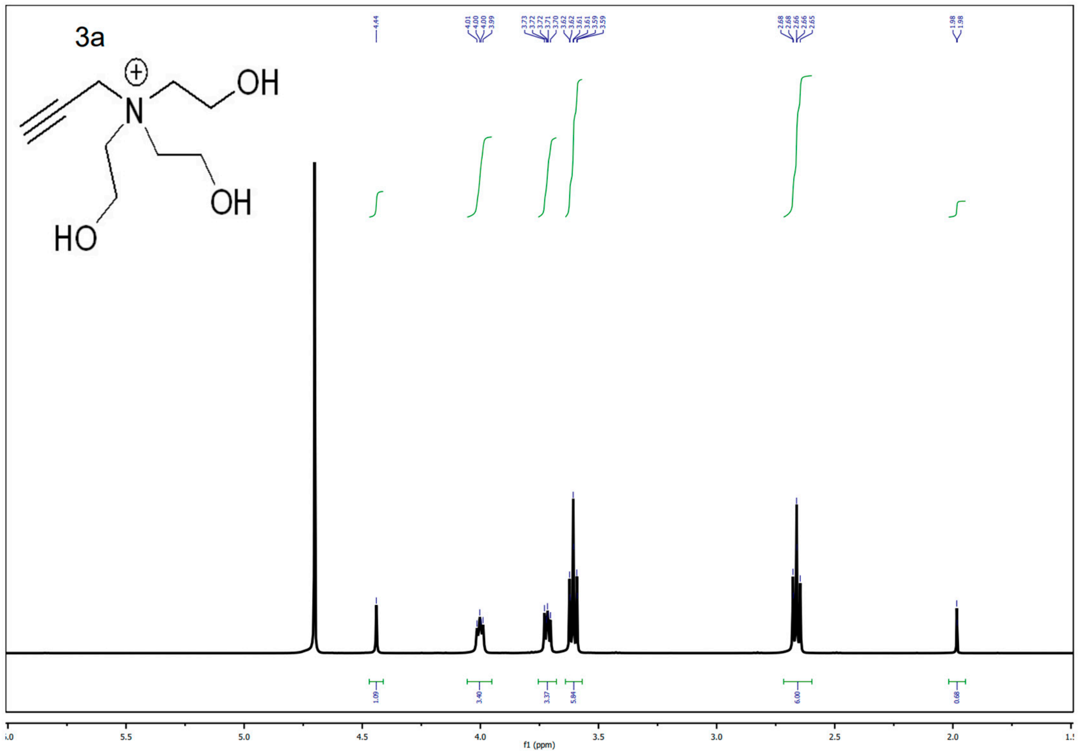

Proton NMR of 4a

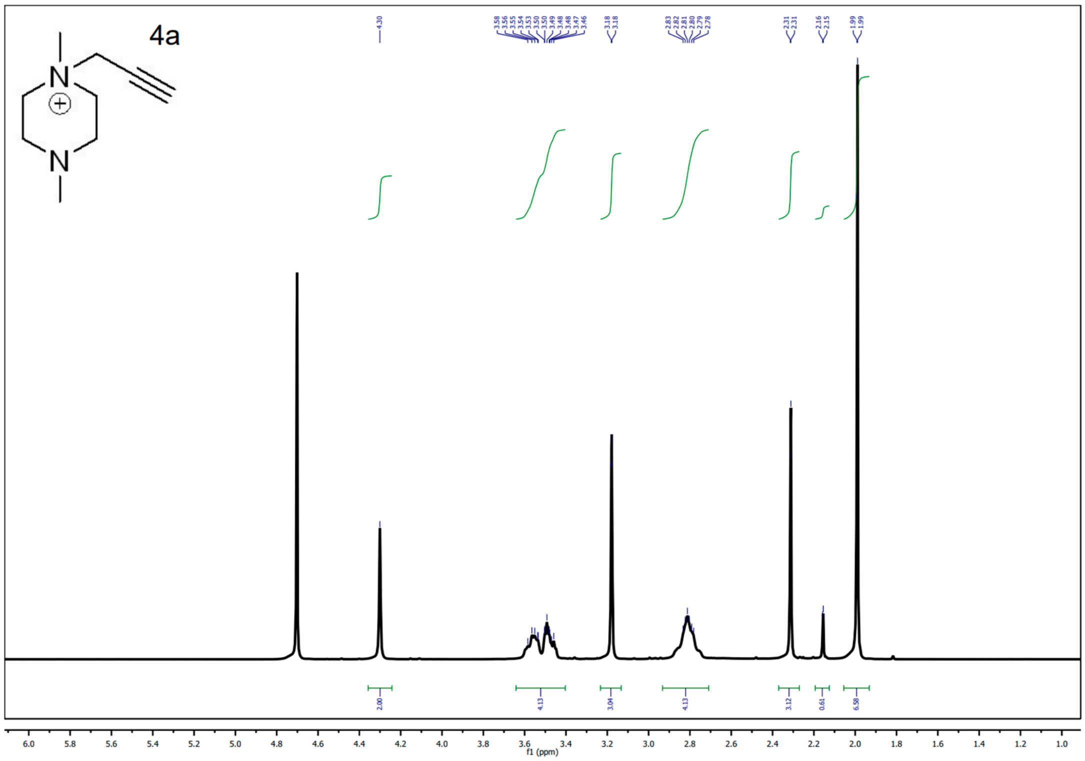

Proton NMR of 5a

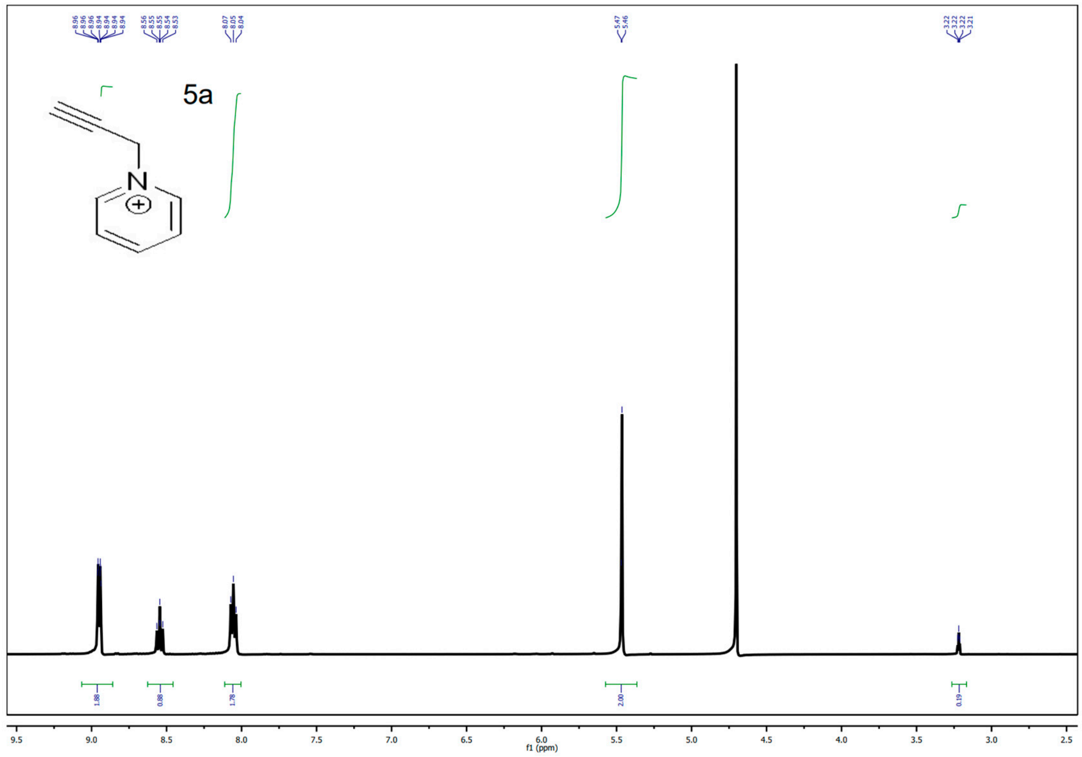

Proton NMR of 6a

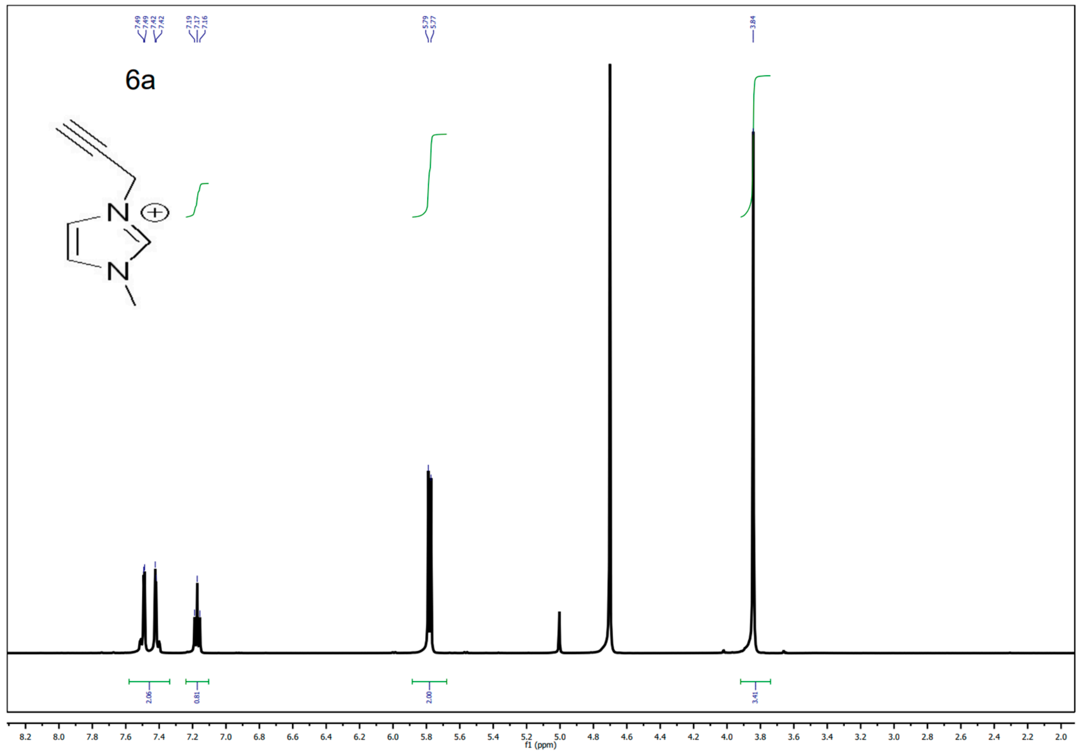

Proton NMR of 7a

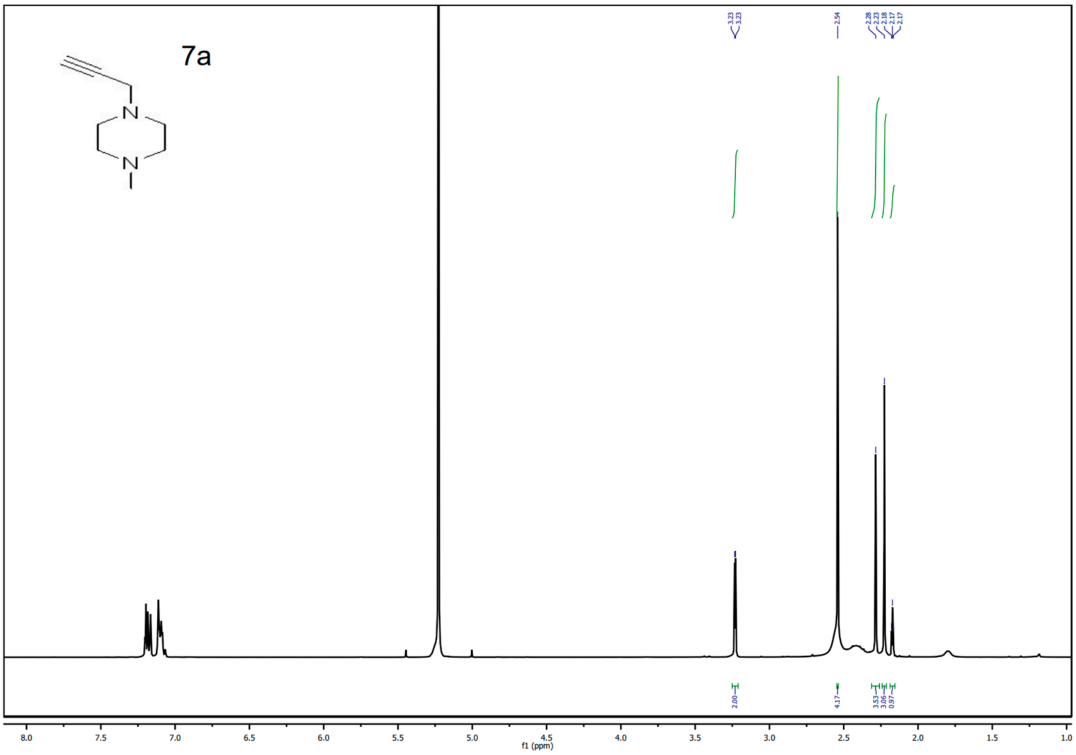

Proton NMR of 8a

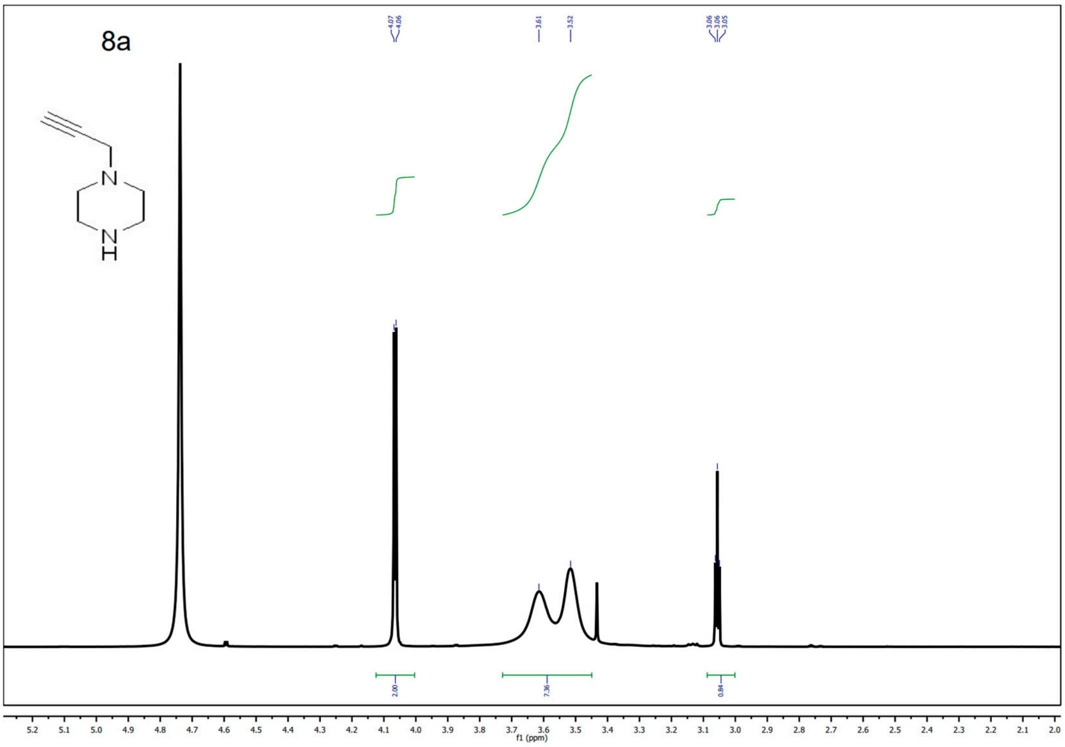

Proton NMR of 9a

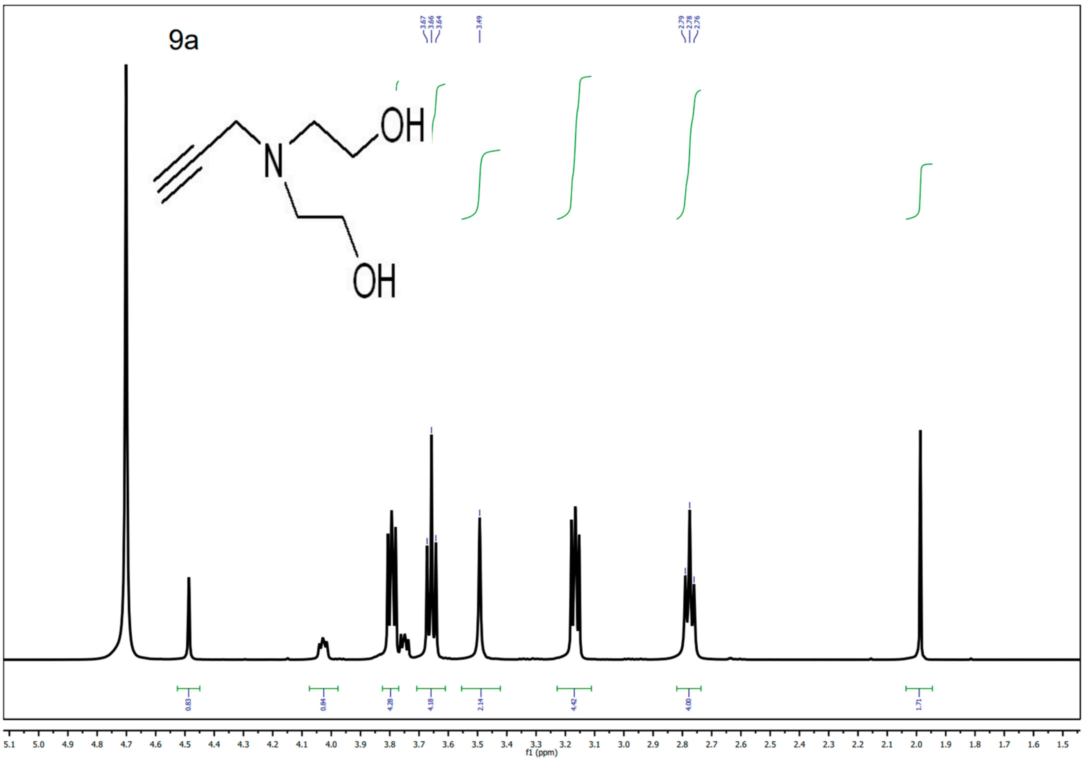

Figure S10. Proton NMR of **1a**–**9a**.

IR Spectra of Chitosan Azide

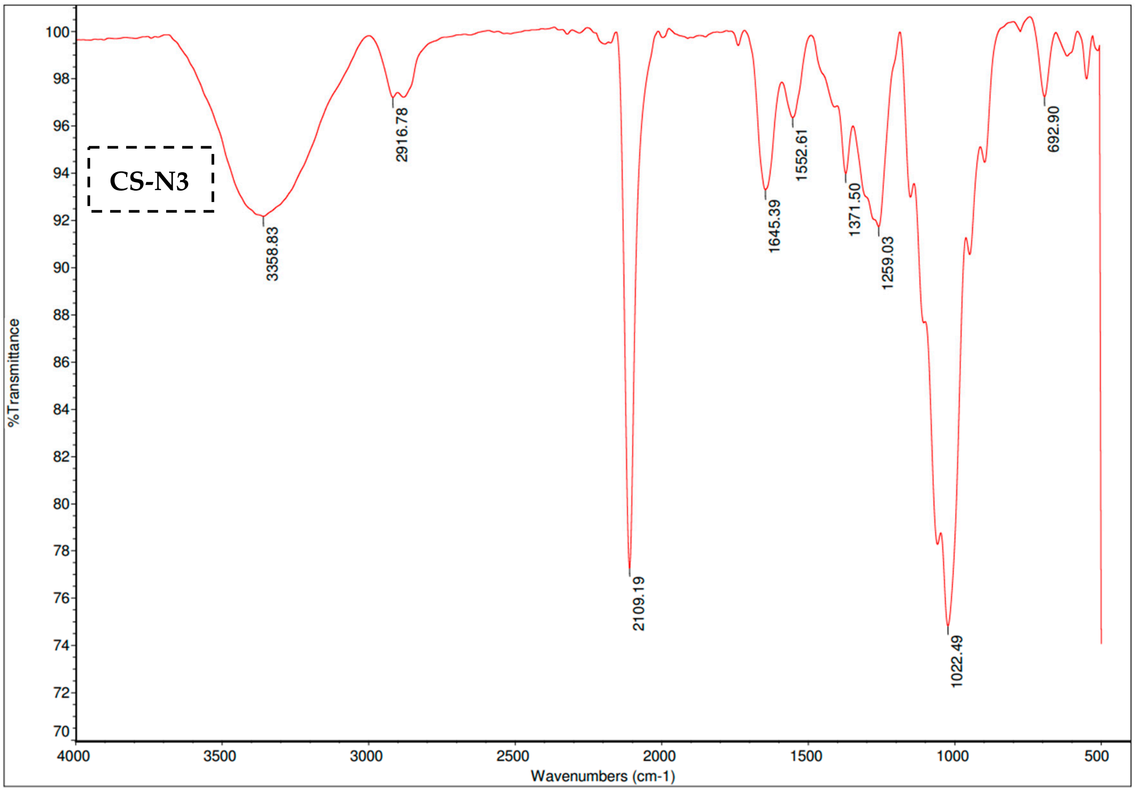

Compound 1

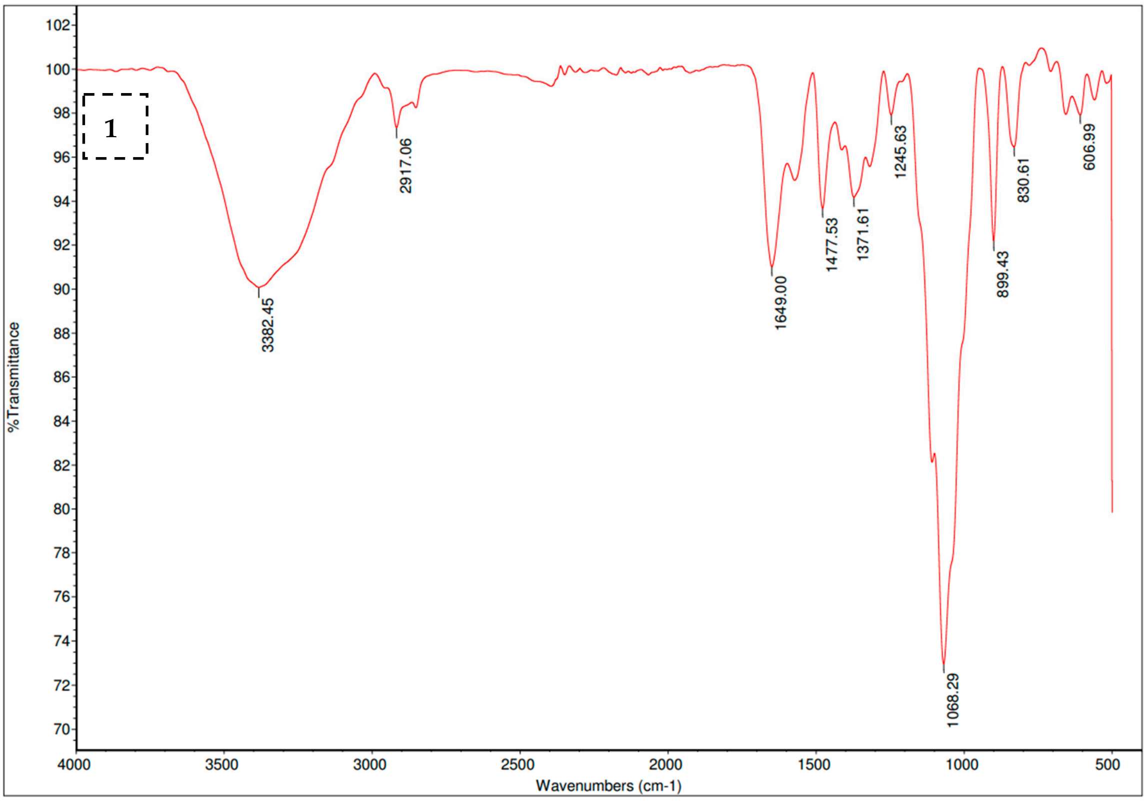

Compound 2

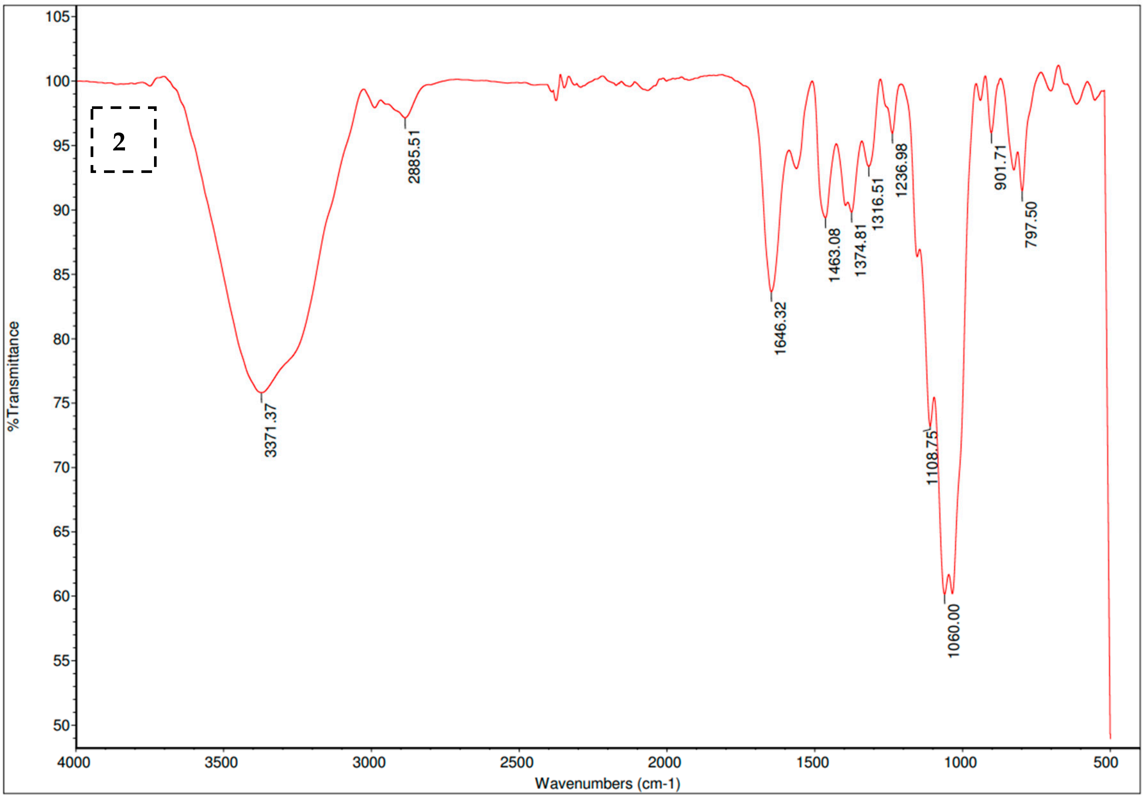

Compound 3

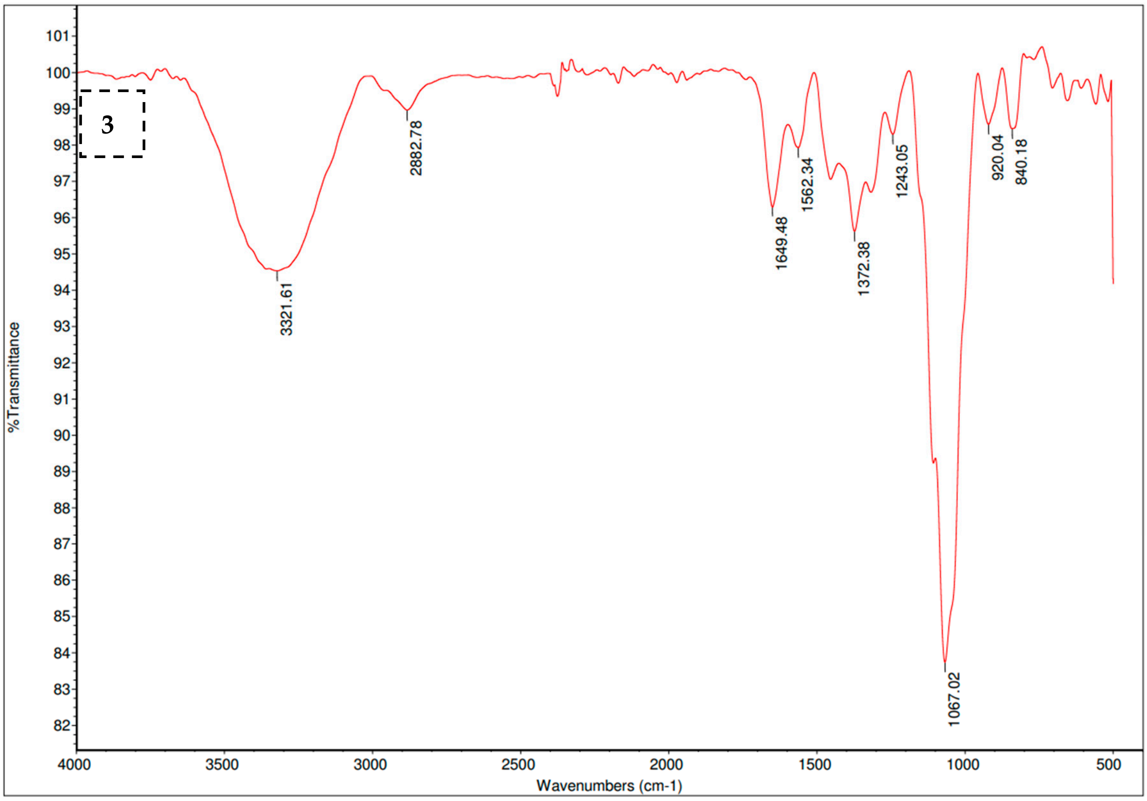

Compound 4

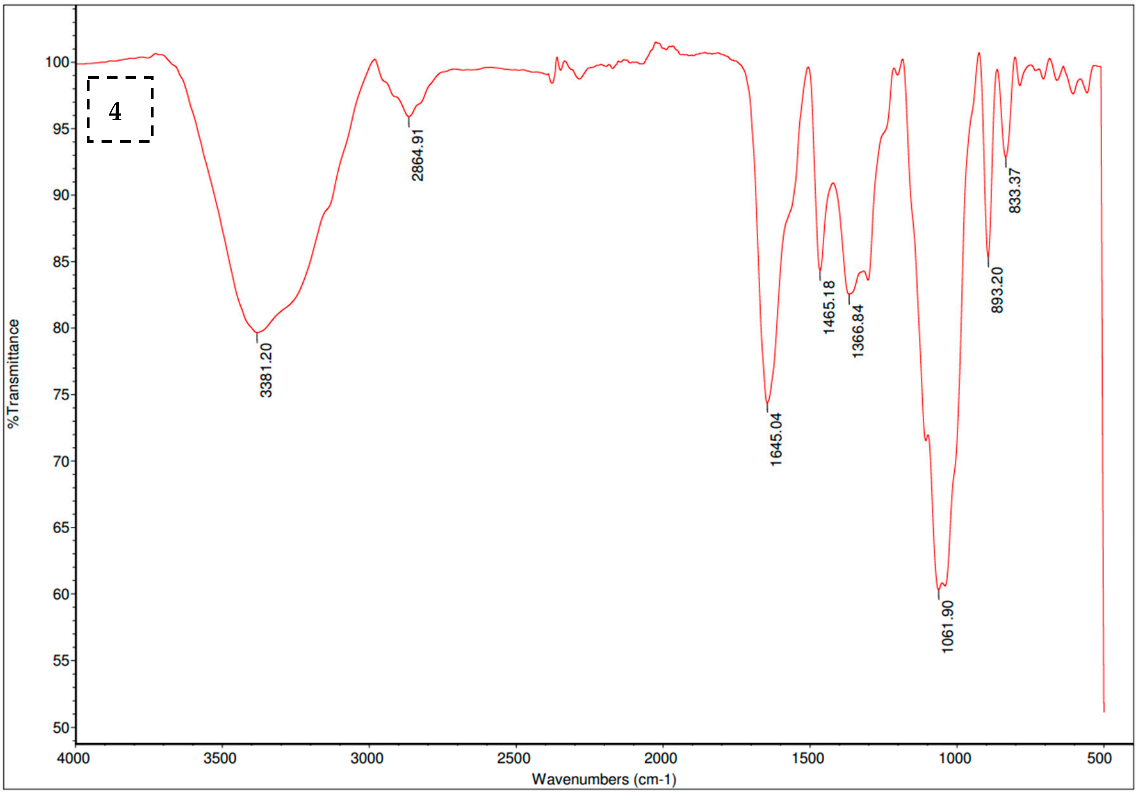

Compound 5

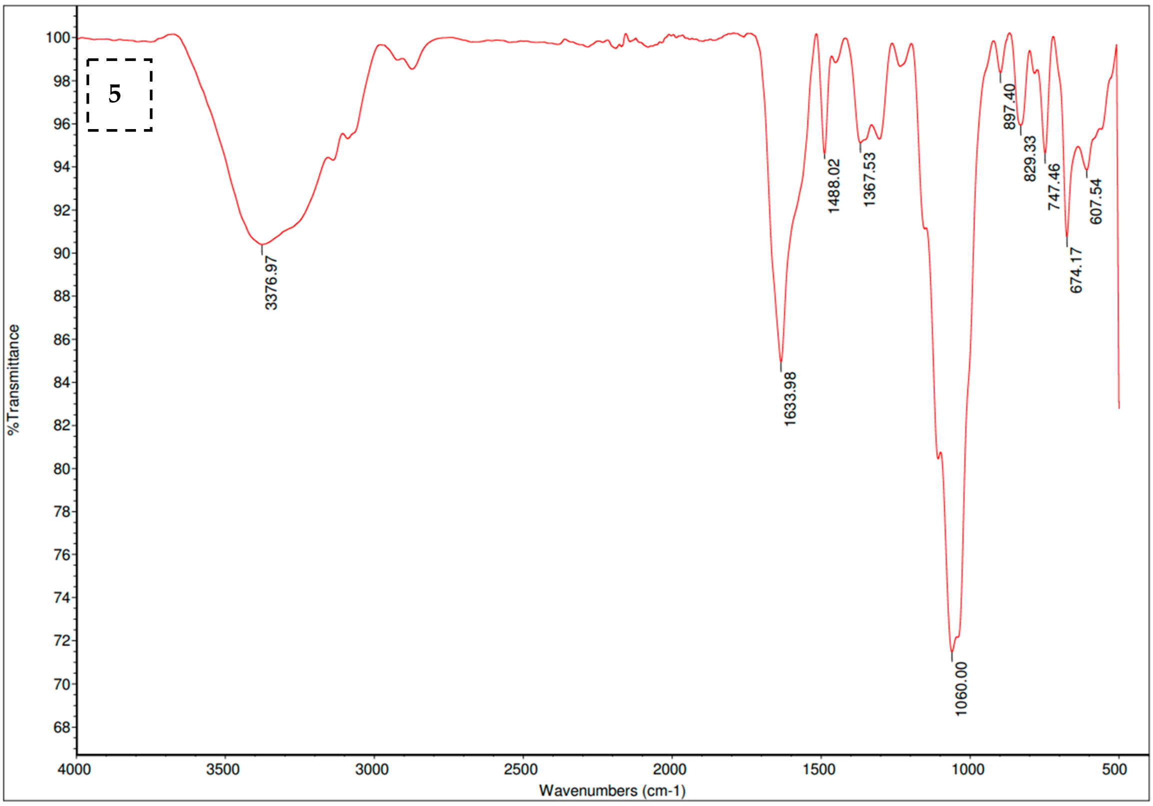

Compound 6

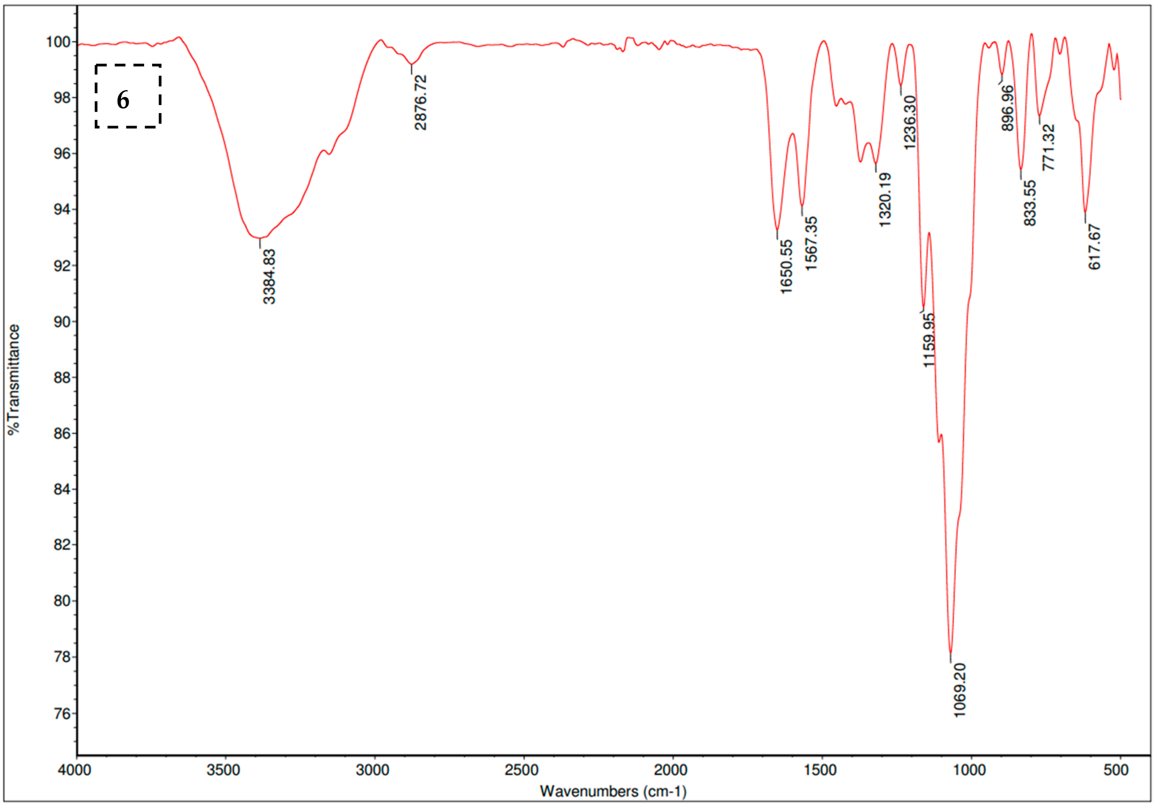

Compound 7

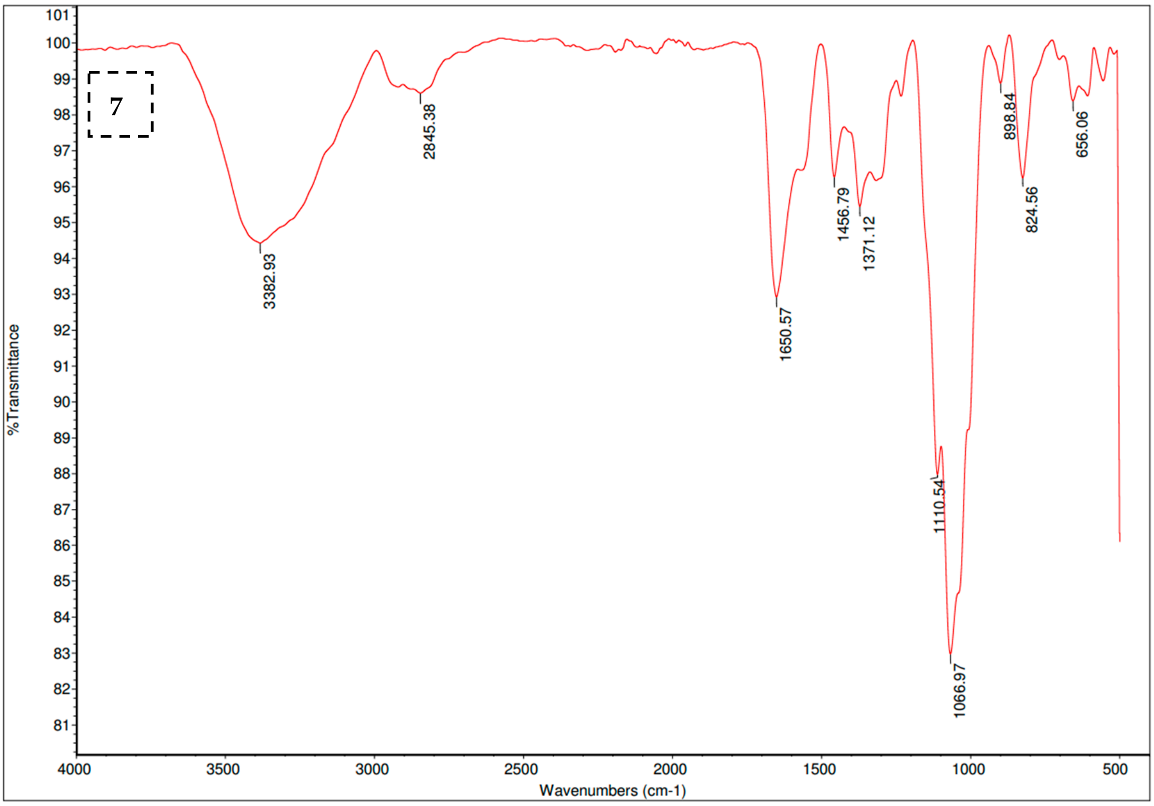

Compound 8

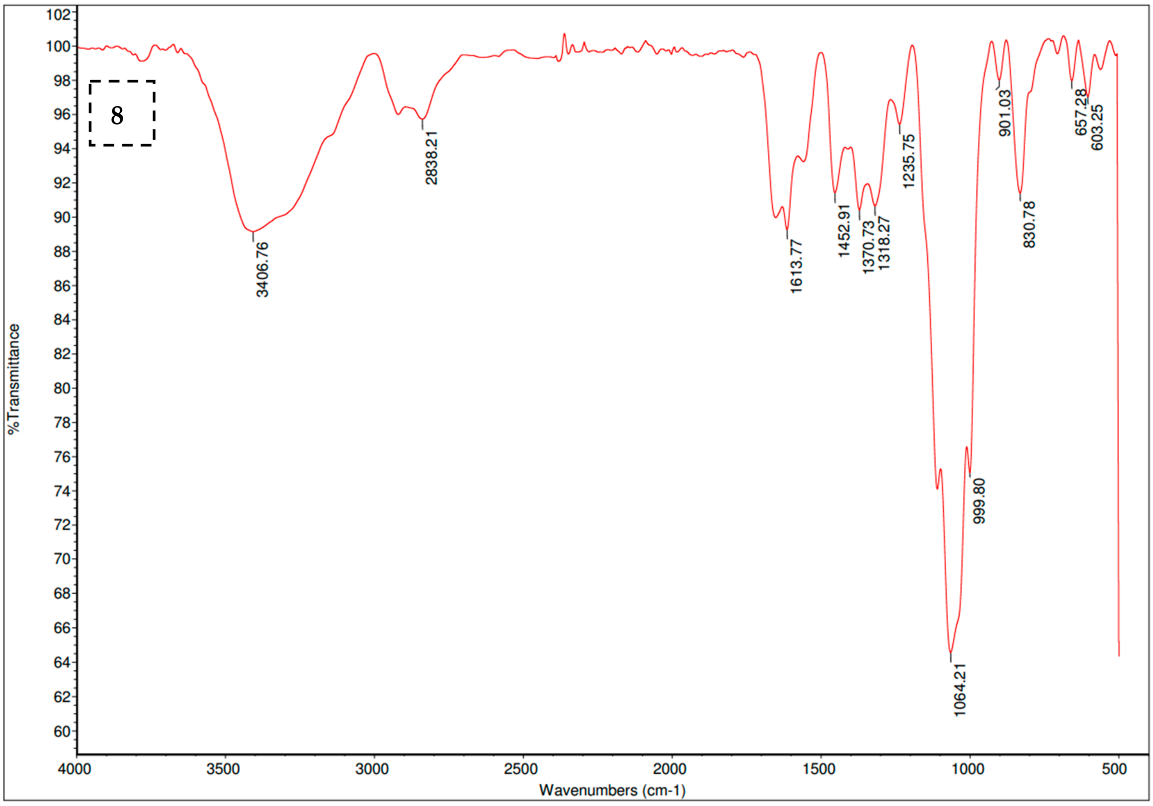

Compound 9

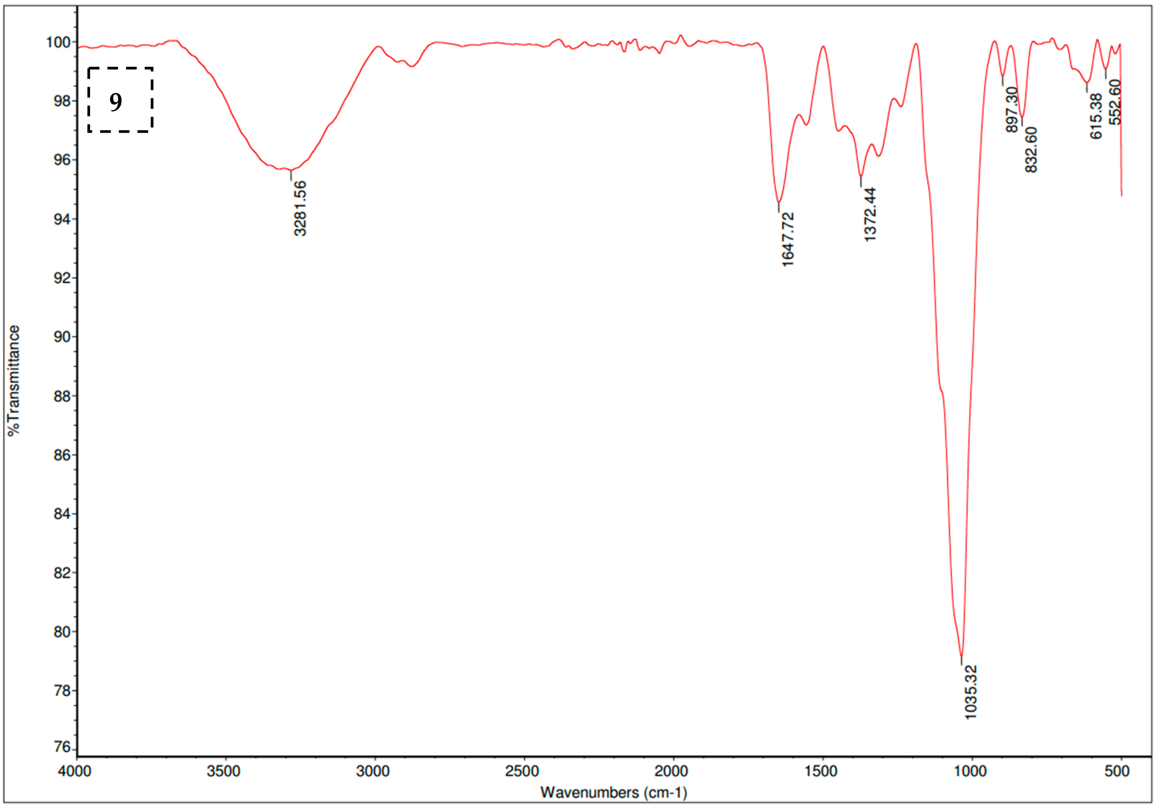

Figure S11. IR Spectra of Chitosan Azide and Compounds **1–9.**
